# Developing new health technologies for neglected diseases: a pipeline portfolio review and cost model

**DOI:** 10.12688/gatesopenres.12817.3

**Published:** 2020-02-19

**Authors:** Ruth Young, Tewodros Bekele, Alexander Gunn, Nick Chapman, Vipul Chowdhary, Kelsey Corrigan, Lindsay Dahora, Sebastián Martinez, Sallie Permar, Johan Persson, Bill Rodriguez, Marco Schäferhoff, Kevin Schulman, Tulika Singh, Robert F Terry, Gavin Yamey

**Affiliations:** 1 Center for Policy Impact in Global Health, Duke Global Health Institute, Durham, NC, 27710, USA; 2 Policy Cures Research, Sydney, NSW, 2010, Australia; 3 School of Medicine, Duke University, Durham, NC, 27710, USA; 4 Duke Human Vaccine Institute, Duke University Medical Center, Durham, NC, 27710, USA; 5 Department of Immunology, Duke University, Durham, NC, 27710, USA; 6 SEEK Developent, Berlin, 10407, Germany; 7 Children’s Health and Discovery Institute; Department of Pediatrics, Duke University, Durham, NC, 27710, USA; 8 Foundation for Innovative New Diagnostics, Geneva, Switzerland; 9 Duke Clinical Research Institute, Duke University, Durham, NC, 27715, USA; 10 Department of Molecular Genetics and Microbiology, Duke University, Durham, NC, 27710, USA; 11 The Special Programme for Research and Training in Tropical Diseases, World Health Organization, Geneva, CH-1211, Switzerland

**Keywords:** research and development, innovation, neglected diseases, global health, financing

## Abstract

**Background**: Funding for neglected disease product development fell from 2009-2015, other than a brief injection of Ebola funding. One impediment to mobilizing resources is a lack of information on product candidates, the estimated costs to move them through the pipeline, and the likelihood of specific launches. This study aimed to help fill these information gaps.

**Methods**: We conducted a pipeline portfolio review to identify current candidates for 35 neglected diseases. Using an adapted version of the Portfolio to Impact financial modelling tool, we estimated the costs to move these candidates through the pipeline over the next decade and the likely launches. Since the current pipeline is unlikely to yield several critical products, we estimated the costs to develop a set of priority “missing” products.

**Results: **We found 685 neglected disease product candidates as of August 31, 2017; 538 candidates met inclusion criteria for input into the model. It would cost about $16.3 billion (range $13.4-19.8B) to move these candidates through the pipeline, with three-quarters of the costs incurred in the first 5 years, resulting in about 128 (89-160) expected product launches.  Based on the current pipeline, there would be few launches of complex new chemical entities; launches of highly efficacious HIV, tuberculosis, or malaria vaccines would be unlikely. Estimated additional costs to launch one of each of 18 key missing products are $13.6B assuming lowest product complexity or $21.8B assuming highest complexity ($8.1B-36.6B). Over the next 5 years, total estimated costs to move current candidates through the pipeline and develop these 18 missing products would be around $4.5B (low complexity missing products) or $5.8B/year (high complexity missing products).

**Conclusions**: Since current annual global spending on product development is about $3B, this study suggests the annual funding gap over the next 5 years is at least $1.5-2.8B.

## Introduction

In 2015, United Nations member states adopted the Sustainable Development Goals (SDGs), an expansive global agenda that includes ambitious health targets. These health targets include, by 2030, “end the epidemics of AIDS, tuberculosis, malaria and neglected tropical diseases,” “end preventable deaths of newborns and children under 5 years of age,” and reduce the global maternal mortality ratio to under 70 per 100,000 live births (see Sustainable Development Goals (SDGs)). Recent studies based on modelling the impacts of scaling up health tools and strengthening health systems show that it is highly unlikely that these targets will be achieved using today’s health technologies alone— achievement will also require breakthrough innovations, such as high efficacy preventive vaccines for HIV, malaria, and tuberculosis ^1– 3^. The Commission on Investing in Health, in its *Global Health 2035* report, also found that achieving “grand convergence”—a reduction in avertable infectious, maternal, and child deaths to universally low levels—will require accelerated health product development ^1^.

Investing in the development and delivery of health technologies is one of the most effective ways to achieve rapid reductions in avertable mortality. For example, Jamison and colleagues recently showed that the diffusion of such technologies accounted for about 80% of the decline in child mortality from 1970 to 2000 ^4^. In addition, researchers have used a new economic evaluation tool termed “extended cost effective analysis” to show that many health technologies for diseases of poverty provide not only health but also financial protection, and are pro-poor ^5^. A further way in which investing in health product development has economic benefits is that the returns to investment can be very large. For example, the March of Dimes invested about US$26 million (M) in developing the polio vaccine, and since routine vaccination was introduced, treatment cost savings have generated a net benefit of around US$180 billion (B) in the United States alone (unless otherwise stated, all dollar figures within this report have been adjusted to 2017 US dollars) ^6^. A Copenhagen Consensus study estimated that every US$1 invested in HIV vaccine development would return US$2-$67, assuming a vaccine of 50% efficacy becomes available by 2030 and annual R&D costs are roughly US$0.9B ^7^.

There is thus a strong case for investing in product development for neglected diseases (in this paper, we use the term “neglected diseases” to refer to the 35 infections or health priorities defined by Policy Cures Research as neglected, including HIV/AIDS, malaria, tuberculosis, pneumonia, diarrheal diseases, neglected tropical diseases and reproductive health needs of developing countries (see G-FINDER project)). Yet funding for neglected disease product development fell steadily from 2009 to 2015, with the exception of a short-term injection of Ebola funding (see G-FINDER report for 2016). While mobilizing additional finance for such R&D is needed, funders face several information gaps that are an impediment to resource mobilization. In particular, there is a lack of consolidated information on:

- which candidates are currently in the pipeline and at what development phase;
- the estimated costs to accelerate this portfolio of candidates to production;
- the anticipated product launches that would result from such acceleration; and
- the critical, highly needed products that would still be “missing” under the status quo.

There have been a small number of studies published on the estimated cost to develop a *single* drug. For example, DiMasi and colleagues estimate that it takes $2.6B (in 2015 US dollars) to develop a new chemical entity (NCE), an estimate based on surveying 10 pharmaceutical firms to obtain information on 106 randomly selected new drugs ^8^. It is unclear how relevant this study is to developing products for neglected diseases, and the estimate can be criticized for, among other things, including $1.2B in “time costs” (the expected returns that private investors forgo while a drug is in development). However, while there are published estimates of the costs of developing individual products, to the best of our knowledge, there have been no estimates of the costs to move the *full portfolio of current product candidates* through the pipeline.

There has been an estimate by the WHO’s Consultative Expert Working Group on R&D (the CEWG) on overall funding needs for neglected disease product development ^9^. The CEWG argued that $6B should be spent annually on such research. This estimate was not based on an empirical analysis of what is in the pipeline, what is missing, and what it would cost to develop the missing tools. Instead, it was derived by doubling the amount that the public sector in low- and middle-income countries invested in health R&D in 2005 (which was $3B, according to the 2008 Global Forum for Health Research report ^10^). There remains a dearth of cost estimates for the actual pipeline of neglected disease candidate technologies.

Our study aimed to help close these information gaps. We conducted a pipeline portfolio review, using public domain knowledge, to examine which candidate products (e.g., drugs, vaccines, diagnostics) are currently in the pipeline for neglected diseases. Based on the results of this review, we estimated the costs to move these candidates through the pipeline, the likely launches, and the “priority” health technologies that would be “missing” under the status quo. To do this, we used an adaptation of a financial tool—the Portfolio to Impact (P2I) model—developed by TDR, the Special Programme for Research and Training in Tropical Diseases, for the World Health Organization ^11, 12^. We made the adaptations of the P2I tool ourselves, resulting in P2I version 2 (P2I v.2); adaptations included adding more product types (e.g., vector control products) and modifying some of the tool’s underlying assumptions based on additional data.

## Methods

In this section, we describe the study’s six steps: (i) pipeline portfolio review; (ii) adaptation of the original P2I model; (iii) classification of candidate health products into archetypes; (iv) inputting the pipeline of candidates into the adapted model; (v) estimating the costs of developing “missing” products; and (vi) sensitivity analysis.

### i) Pipeline portfolio review

**Scope.** A global review of candidate products for neglected diseases was conducted by Policy Cures Research (authors NC and VC) in 2017, and represents a snapshot of the pipeline as of August 31, 2017. Policy Cures Research, a non-profit global health research and advocacy organization, developed the scope of the R&D pipeline presented in this analysis based on the G-FINDER landscape reports on global funding of R&D for neglected diseases and the reproductive health needs of developing countries. The G-FINDER scope is based on three key principles: the disease or health issue disproportionately affects the developing world; there is a need for new products (i.e., there is no existing product, or improved or additional products are needed); and there is market failure (i.e., there is insufficient commercial market to attract private R&D investment). Accordingly, the R&D pipeline presented here encompasses the 33 neglected diseases included in the G-FINDER report, as well as developing country-specific reproductive health needs, and Ebola, giving a total of 35 neglected diseases or conditions.

Product types included were drugs, vaccines, diagnostics, vector control products, contraceptives, and multipurpose prevention technologies (MPTs); however, in line with the G-FINDER scope, not all product types were included for all areas. Microbicide candidates for HIV have been included under the drug category, while contraceptives and MPTs (which prevent pregnancy *and* sexually transmitted infections) have been grouped as reproductive health technologies. Medical devices (except for diagnostics and contraceptives), and general or supportive therapies (e.g., oral rehydration or nutritional supplements), were excluded.

We define the pipeline to include product candidates at all stages of development—from discovery through product registration. For drugs, vaccines, and reproductive health products, development stage was broken down into discovery; pre-clinical studies; and clinical trials (further divided into Phase I, Phase II and Phase III trials). Diagnostics and vector control products have different product development and regulatory pathways, and the development stage for these products was broken down into concept and research; feasibility and planning; design and development; and clinical validation and launch readiness. Early stage drug discovery projects that are not linked to a specific, discrete pipeline candidate were excluded. Candidates were no longer considered to be part of the R&D pipeline—and therefore were excluded from this analysis—once granted regulatory approval by a national regulatory authority, or if their development had been placed on hold indefinitely.

In line with the scope of the G-FINDER report, additional restrictions were applied to selected disease and product categories with potential commercial (high-income country market) overlap. For example, drug candidates for HIV/AIDS were only included if they were label-extensions or reformulations specifically intended for developing country use (e.g., paediatric or slow-release formulations; fixed dose combinations; or low-dose formulations for prophylaxis).

Further details on the specific diseases and related product areas within the scope of the pipeline analysis are given in the Policy Cures Research R&D matrix and R&D scope document. Additional details about the search are in the online methodology.

**Data sources and validation.** Policy Cures Research collected data on the candidates in the global R&D pipeline for 35 neglected diseases. This new 2017 product portfolio review built on Policy Cures Research’s previous pipeline analysis conducted (as Policy Cures) in 2015, pipeline data collected for the 2012 Policy Cures/Global Health Technologies report *Saving Lives and Creating Impact: Why Investing in Global Health R&D Works*, and the BIO Ventures for Global Health (BVGH) Global Health Primer.

To bring the R&D pipeline candidate data up to date as of August 31, 2017, and to expand on the scope of previous efforts, Policy Cures Research reviewed and cross-referenced all major sources of available data on the R&D pipeline for poverty-related and neglected diseases. Sources included the G-FINDER R&D funding database; the World Health Organization “ rainbow tables”; background documents prepared for the WHO Product Development for Vaccines Advisory Committee (see WHO page of Immunization, Vaccines and Biological R&D); WHO Vector Control Advisory Group (VCAG) reports; WHO Pesticide Evaluation Scheme (WHOPES) reports; Unitaid landscape and technical reports; disease-specific pipeline updates prepared by BVGH and the Treatment Action Group; publicly available company and product development partnership R&D portfolios; journal publications; clinical trial registration portals; and university, government, and non-profit organization websites.

Candidates were only included if an authoritative source could confirm they were still in active development. The following sources were considered to be authoritative:

- The website of the candidate developer, if recently updated
- Recent reports or other materials from international organizations such as WHO and Unitaid
- Clinical trial portals
- Correspondence with product developers
- Correspondence with experts in the field, including FIND; the Innovative Vector Control Consortium (IVCC); the International AIDS Vaccine Initiative (IAVI); Netherlands Leprosy Relief; Program for Appropriate Technology in Health (PATH); the Sabin Vaccine Institute; and the US National Institute of Allergy and Infectious Diseases (NIAID).

### ii) Development of the P2I v.2 costing model

In order to estimate the costs of moving these candidate health products for neglected diseases through the pipeline, and the likely resulting product launches, we made adaptations to an existing, custom-built costing model, P2I (we call the original model P2I version 1, or P2I v.1). We call the adapted model P2I version 2 (P2I v.2).

**Figure 1.**
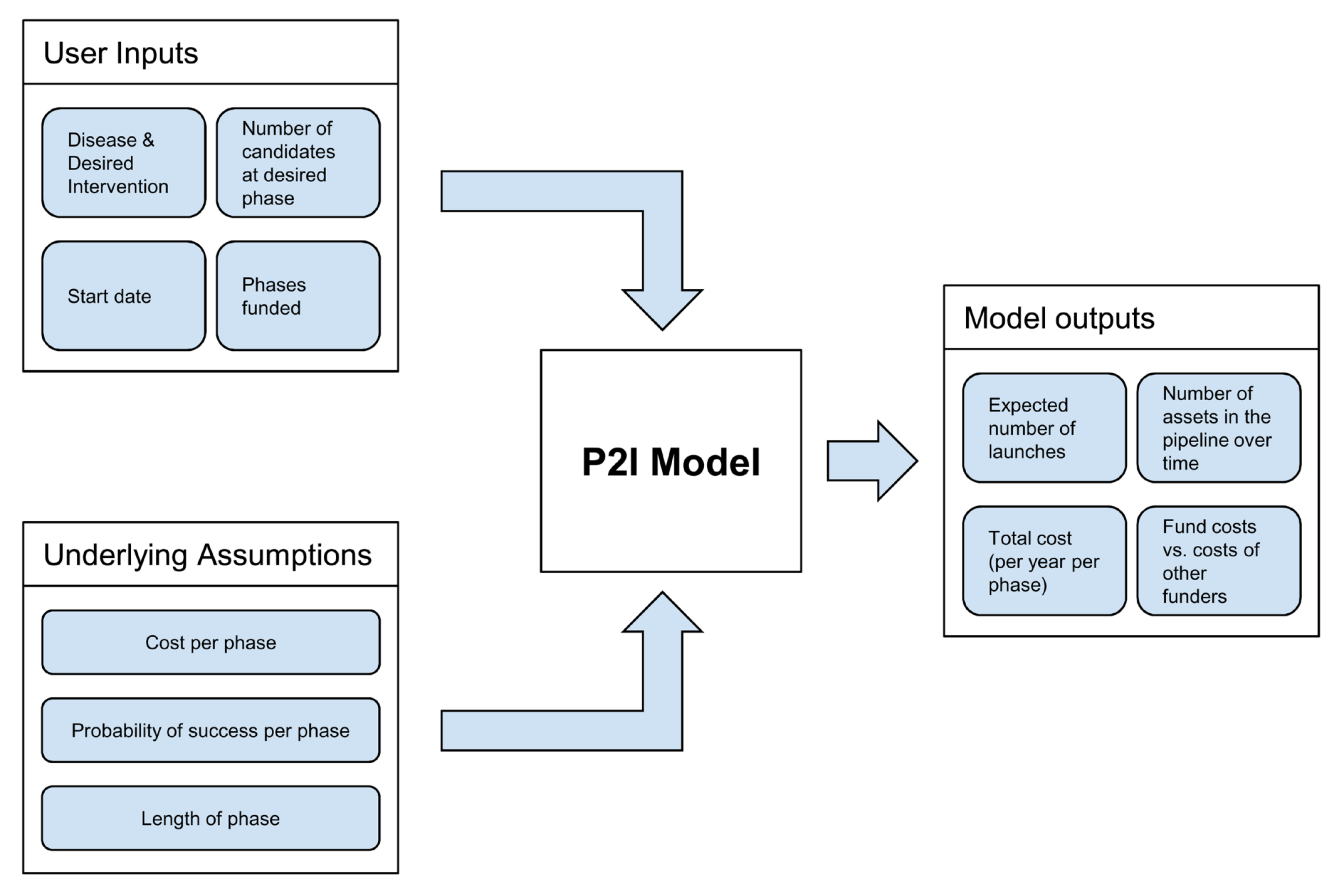
Conceptual overview of P2I model.

The P2I v.1 tool is a user-friendly, public Microsoft Excel file (see Supplementary with reference 12) with costing assumptions and formulae built in ^12^. The tool was developed by TDR. In brief, P2I v.1 is a financial portfolio model that estimates funding needs to move a *portfolio* of candidate health products through the pipeline from late stage pre-clinical to phase III clinical trials, as well as potential product launches over time ( Figure 1). The model, which is deterministic, is based on assumptions for costs, attrition rates (probability of success), and cycle times for four development phases (preclinical to phase III) for eleven different kinds of medical products, called *archetypes* ( Table 1). A detailed description of how these assumptions were developed is given in the accompanying study on development of the P2I v.1 tool ^12^.

**Table 1.** Descriptions of archetypes from the P2I v.1 model.

Archetype		Description	Examples
Vaccine	Simple	Platform has been used to develop other vaccines	Hepatitis A, hepatitis B, polio
	Complex	Requires completely novel approach; no platform; no existing research	Pneumococcal conjugate vaccine (PCV), meningitis B
New Chemical Entity (NCE)	Simple	Validated target or mechanism of action	Primaquine
	Innovative	Novel target or mechanism of action with understanding of disease pathogenesis	Ibrutinib
	Complex	Novel target or mechanism of action without understanding of disease pathogenesis	Imatinib
Repurposed Drug	Simple	Drug has sufficient safety data to start development in Phase II	Azithromycin, doxycycline
	Complex	Drug requires some Phase I clinical trials to verify safety in humans	Moxidectin
Biologic	Simple	Validated target or mechanism of action	IL-17 antibody
	Complex	Novel target or mechanism of action	Natalizumab
Diagnostics	Assay development	Development of a diagnostic assay	Lateral flow tests, quantitative molecular tests
	Simple technical platform development	Development of a technological platform that enhances current technology	Ultrasensitive malaria rapid diagnostic test (RDT)

In brief, the assumptions on development costs at each phase of product development were initially based on a bottom-up analysis of clinical trial costs from Parexel’s R&D cost sourcebook ^13^. The assumptions on attrition rates and cycle times at each phase were initially based on a review of the attrition rates and cycle times of more than 25,000 development candidates. These assumptions were further refined and validated based on (i) academic literature ^14^, (including literature on the development of antibody therapeutics) (ii) industry publications and databases (such as Pharmaprojects), and (iii) stakeholder interviews with a wide variety of PDPs, biopharmaceutical and diagnostic companies, and major funders of global health R&D. For the stakeholder interviews, a total of 228 stakeholders representing a cross-section of the global R&D landscape were contacted to request an interview and 133 agreed to be interviewed, a response rate of 58% (see reference 11 for further details). Overall, the stakeholder interviews largely confirmed the validity of the assumptions derived from the R&D cost sourcebook, and did not lead to any significant changes. As a final validation step, the P2I model and its assumptions were reviewed by TDR’s Science and Technical Advisory Committee, who provided an additional round of expert inputs.

The P2I v.1 tool allows users to make multiple types of adaptations. For example, (i) users can add archetypes; (ii) users can input disease-specific assumptions (e.g., the attrition rates in developing TB biologics); and (iii) if users have their own additional data on costs, attrition rates, and cycle times across the portfolio, they can enter the tool and modify the assumptions. For this study, we made all three types of adaptations.

First, we wanted to add archetypes that were not in version 1, such as vector control products and unprecedented vaccines (discussed further below). Second, we wanted to refine the model as it applied to a specific set of TB candidate archetypes. Third, we took the opportunity to modify a small number of the assumptions. These three adaptations were made based on data shared by the Bill & Melinda Gates Foundation (Per Liljenberg, personal communication). The Foundation supported the development of the P2I v.1 tool, and has used the tool to estimate its own portfolio development costs, but it has made a number of adaptations derived from three Foundation costing exercises:

- In 2010, the Foundation identified, reviewed, and costed out all projects in its product development portfolio, building a portfolio model (including costs, time, and risk) called the Risk-Adjusted Portfolio (RAP) Model, based on publicly available data as well as from a proprietary database. The probability of technical success was based on an attrition database, which contains detailed attrition rates across more than 3,000 products over a 10–20 year period. Additionally, PDPs and industry experts were engaged to identify appropriate ranges for specific programs.
- This costing exercise was repeated in 2012–2013.
- In 2016, the costing exercise was repeated once more, using data compiled from various sources, including from the Foundation itself, from the 2010 RAP Model, and from TDR (the data used in P2I v.1).

Below we summarize the adaptations in the P2I v.2 model, which are based on outputs of the 2016 exercise.

The P2I v.2 model has two additional archetypes, “unprecedented vaccines” and “other products”:

- **Unprecedented vaccines.** In addition to sub-classifying vaccine candidates into “simple” and “complex,” we added “unprecedented vaccines” as a third vaccine candidate category. We assigned candidate vaccines for HIV, TB, and malaria to this third sub-category, which we considered as unprecedented as current platforms have not led to suitable vaccines. We assumed that vaccines targeting each of these diseases would require the development of innovative platforms and a better understanding of the basic biology and of immune protection. The assumptions for unprecedented vaccines were provided by the Bill & Melinda Gates Foundation, which used the P2I v.1 assumptions for complex vaccines as a starting point. As shown in Table 2, the assumptions for unprecedented vaccines are the same as for complex vaccines, with two exceptions. In the P2I v.2 model, unprecedented vaccines have a lower probability of success in phases II and III than complex vaccines (in phase II, 5% for unprecedented vaccines versus 22% for complex vaccines; in phase III, 40% probability of success for unprecedented vaccines versus 64% for complex vaccines). These probability rates for unprecedented vaccines (5% in phase II, 40% in phase III), provided by the Bill & Melinda Gates Foundation, were based on around 10–25 data points per estimated value (these data were from both the Foundation portfolio and publicly available sources). These lower probabilities of success compared with complex vaccines reflect the Foundation’s real world experience of trying to develop highly effective vaccines against HIV, TB, and malaria.
- **Other products**: We used the “other products” archetype for vector control products (which were not included in P2I v.1). The assumptions on costs, attrition rates, and cycle times per phase for the “other products” archetype were derived from product development data shared by the Bill & Melinda Gates Foundation and from the RAP Model.

In addition to including these two archetypes, we also modified several assumptions, as follows:

- **The costs of phase III for simple and complex vaccines.** We used an assumption of $201M for simple vaccines and $223M for complex vaccines. These phase III cost assumptions, which are higher than those in the P2I v.1 model (which assumes a cost of $111.1M for simple vaccines and $133.3M for complex vaccines), were derived from the RAP model. The cost assumptions are higher in the RAP model because they include manufacturing costs of around $90M.
- **The probability of success at phase III for simple NCEs for TB and simple biologics for TB.** For TB candidates in phase III for these archetypes, we used a lower probability (50% for simple NCEs for TB and 55% for biologics for TB) than the probability used in the P2I v.1 model (70% for both simple NCEs for TB and for biologics for TB). These lower probabilities, which are more conservative than those in P2I v.1, are based on an expert assessment of the limited existing data on TB product development, an assessment conducted by the Bill & Melinda Gates Foundation.
- **Assumptions on biologics across the portfolio of diseases.** The cost, probability of success, and cycle time for simple and complex biologics for all diseases at all stages were adapted based on data from Bill & Melinda Gates Foundation (the costs of Phase III for both archetypes included input from P2I v.1 parameters and from the Bill & Melinda Gates Foundation). An assessment of benchmarks for biologics was made by Foundation experts, and could be classified as cautious expert judgement based on early industry trends. The Foundation judged that since most early successful monoclonal antibody projects were related to the tumor necrosis factor alpha pathway but biologics are now finding a much broader range of biological targets, the success rates are likely to fall. This was a prediction based on early signals from industry reporting challenges to time, cost and success rates in many ongoing programs at the time, rather than an assessment based on actual program failures.
- **Development phases for diagnostics.** For diagnostics, the selection and validation phase was further sub-divided into two sub-phases: “concept and research” followed by “feasibility and planning” (see section (iii) for further details).

Table 2 shows the assumptions that we used in P2I v.2.

**Table 2.** Assumptions on costs, attrition rates, and cycle times per phase used in the P2I v.2 model.

Archetype	Cost per phase ($, millions)				Length of phase (years)				Probability of success (%)			
	Preclinical	Phase 1	Phase 2	Phase 3	Preclinical	Phase 1	Phase 2	Phase 3	Preclinical	Phase 1	Phase 2	Phase 3
Simple vaccine	6.7	2.2	13.2	201.0	3.4	1.6	2.2	2.3	41.0	68.0	46.0	71.0
Complex vaccine	16.6	2.5	13.9	223.0	3.3	2.0	3.7	3.5	41.0	50.0	22.0	64.0
Unprecedented vaccine	16.6	2.5	13.9	223.0	3.3	2.0	3.7	3.5	41.0	50.0	5.0	40.0
Simple NCE	5.0	2.2	5.8	32.8	2.5	1.8	3.4	3.2	65.0	60.0	39.0	69.0
Simple NCE for TB	5.0	2.2	5.8	32.8	2.5	1.8	3.4	3.2	65.0	60.0	39.0	50.0
Complex NCE	10.0	7.4	6.4	36.1	2.9	1.9	3.5	2.8	55.0	57.0	20.0	40.0
Simple repurposed drug	5.0	2.2	5.8	17.6	2.3	1.6	2.1	2.1	75.0	59.0	46.0	68.0
Complex repurposed drug	5.0	2.2	5.8	17.6	2.3	1.6	2.1	2.1	75.0	59.0	46.0	68.0
Simple biologic	6.7	2.2	13.2	122.0	3.4	1.6	2.2	3.1	41.0	68.0	46.0	70.0
Simple biologic for TB	6.7	2.2	13.2	122.0	3.4	1.6	2.2	3.1	41.0	68.0	46.0	55.0
Complex biologic	16.6	2.5	13.9	126.0	3.3	2.0	3.7	2.8	41.0	50.0	22.0	40.0
	Concept and Research	Feasibility and Planning	Design and Development	Clinical Validation and Launch Readiness	Concept and Research	Feasibility and Planning	Design and Development	Clinical Validation and Launch Readiness	Concept and Research	Feasibility and Planning	Design and Development	Clinical Validation and Launch Readiness
Diagnostic, assay development	1.5	1.5	2.0	3.5	0.5	0.5	2.0	1.3	71	71	100	100
Diagnostic, simple platform development	1.5	1.5	100.0	3.5	0.5	0.5	2.5	2.0	71	71	75	100
Other products	1.2	1.2	1.1	2.6	0.5	0.5	1.0	1.0	87	87	95	80

Sources of data:
P2I model assumptions	McKinsey RAP	Bill & Melinda Gates Foundation

Finally, P2I v.1 allows users to input a portfolio of up to 150 product development projects (the model was developed in the context of potentially launching a pooled fund for R&D that would have had capacity constraints). However, the pipeline of candidates for neglected diseases has far more than 150 projects, so we adapted the tool to be able to input all of these projects.

### iii) Classification of product candidates into archetypes

As a starting point, we used the initial archetypes and the descriptions of each archetype from P2I.v1 ( Table 1). In order to further “operationalize” each archetype—i.e., to make it easier for the research team to make decisions about how to classify each candidate—we worked with technical experts to further define each archetype. Table 3 shows the original archetype descriptions from P2I v.1, our additional definitions, and examples of candidate classifications.

**Table 3.** Further definitions of archetypes to guide classification of product candidates.

Product archetype		Description from P2I v.1 model	Examples	Further definition
**Repurposed** **drug**	Simple	Drug has sufficient safety data to start development in phase II	azithromycin, doxycycline	Any drug that has already been approved for market use in humans and is now being used in a new formulation or for a new indication.
	Complex	Drug requires some phase I clinical trials to verify safety in humans	moxidectin	Any drug that has already been approved for market use in humans and still requires clinical trials to verify safety in humans.
**New** **chemical** **entity (NCE)**	Simple	Validated target/mechanism of action	primaquine	Any chemically synthesized drug that is part of a well-established class of drugs or has a mechanism of action that has already been approved for market use in humans. It is not the first of its class to be approved.
	Complex	Novel target/mechanism of action without understanding of disease pathogenesis	imatinib	Any chemically synthesized drug that is first in its class as determined by having a novel target/mechanism of action regardless of the drug’s indication.
**Biologics**	Simple	Validated target/mechanism of action	human monoclonal antibody m102.4	Any drug that is synthesized from a living organism that is part of a well- established class of drugs or has a mechanism of action that has already been approved for market use in humans. It is not the first of its class to be approved.
	Complex	Novel target/mechanism of action	polyclonal IgG antibodies	Any drug that is synthesized from a living organism that is first in its class as determined by having a novel target/mechanism of action regardless of the drug’s indication.
**Vaccines**	Simple	Platform has been used to develop other vaccines.	Hep A, Hep B, polio, killed or live attenuated vaccines	Any vaccine platform that has been extensively researched and approved for use in the past. Pathogen has readily-identifiable vaccine targets that lack complexity. Conferral of immunity against disease-causing microorganism is expected as natural immunity to the pathogen is protective. Platform is likely to elicit robust protective response.
	Complex	Requires completely novel approach; no platform; no existing research	Pneumococcal conjugate vaccine, meningitis B, DNA or mRNA vaccines	Any vaccine platform that requires a novel approach that has not been successfully approved for use in the past. Conferral of immunity against disease-causing microorganism is difficult to induce and maintain and natural immunity is not protective against reinfection. Platform may elicit incomplete/insufficient immunity and require boosting over time.
	Unprecedented	Defined in the same way as complex vaccines, but with lower probabilities of success	HIV, TB, malaria vaccines	All vaccine candidates for HIV, TB, and malaria are classified as “unprecedented” due to much higher attrition rates at phases II and III than other complex vaccines
**Other** **products**		This category refers to vector control products	Long-lasting insecticide-treated bed nets, new chemical pesticides	Chemical pesticides intended for global public health use that aim to control/kill vectors associated with transmitting poverty-related diseases; biological control interventions that aim to control/kill vectors associated with transmitting poverty-related diseases; veterinary vaccines designed to prevent animal-to-human transmission of neglected diseases.
**Diagnostics**	Assay development	Development of a diagnostic assay	Lateral flow tests, qualitative/ quantitative molecular tests	Any new diagnostic that represents menu extension on an existing platform with an assay targeting a neglected disease.
	Simple technical platform development	Development of a technical platform that enhances current technology	Ultrasensitive malaria rapid diagnostic test	Any new diagnostic that relies on a novel approach to sample processing or target detection.

As shown in Table 3, product candidates were classified into six broad archetypes—repurposed drugs, NCEs, vaccines, biologics, diagnostics, and “other products” (which refers to vector control products). Repurposed drugs, NCEs, and biologics were further sub-classified into simple versus complex; vaccines into simple, complex, or unprecedented; and diagnostics into assay development versus simple technical platform development. For candidates in the pipeline that were contraceptives, microbicides, or MPTs, these were classified according to the constituent drug (e.g., microbicides in the pipeline were classified as repurposed drugs, NCEs, or biologics). If there was more than one active drug ingredient in the MPT, the candidate was classified according to the most complicated component. We did not consider if the polymer or technology itself was innovative in itself as this went beyond the scope of our costing framework.

The classification of candidates was made by different members of the research team, based on their expertise (repurposed drugs, NCEs, and biologics: KS, KC; diagnostics: BR; vaccines: SP, LD, TS; other products: VC). The classification was based on a combination of (a) technical expertise of the researchers, (b) academic literature, (c) relevant publicly available product databases (e.g., for classifying drugs, ChemBL and chemspider), (d) information from international clinical trials registries, including the WHO International Clinical Trials Registry Platform, (e) websites of PDPs, e.g. the Medicines for Malaria Venture website, (e) patent databases, and (f) relevant reports and news releases from bilateral and multilateral funding agencies, companies, PDPs, other product developers, and non-government organizations. In assigning each candidate product to an archetype, we documented any relevant source material that guided the classification (e.g., a published research article on the candidate’s mechanism of action).

Classification of candidate diagnostics into archetypes was conducted by a technical expert in diagnostics R&D (BR). Using the Policy Cures Research list of candidates, these were further classified into six more specific development phases, as required by P2I v.2: concept; feasibility; early development; late development; validation; and commercialization. Table 4 summarizes what these phases mean and how they compare with two other classification systems for technology readiness (the Technology Readiness Level (TRL) and Manufacturing Readiness Level (MRL), developed by the United States Department of Defense). When it came to inputting candidates into the adapted P2I cost model (as shown in Table 2), diagnostic candidates at the concept phase were placed in the category “concept and research”; those in the feasibility stage were placed in “feasibility and planning”; those in either early or late development were placed in “design and development”; those in validation were placed in “clinical validation and launch readiness”; and those in the commercialization phase were excluded from the cost modelling.

**Table 4.** Our classification system for classifying diagnostic candidates into phases.

Broad categories provided by Policy Cures Research	Stage	Our classification system	TRL equivalent	MRL equivalent	Short descriptor	Description of stage	Milestone at end stage	Status of risk assessment	Status of data	Status of quality management system
**Early** **Development**	**Selection and** **Validation**	**Concept**	**1**	**--**	*What is the* *idea?*	Technical concept (whether innovative mechanism or unique integration of proven concepts) is under investigation.	Concept design, preliminary data	Technical risks, manufacturing risks, business risks not yet fully known.	Preliminary data on low *n* evaluations may be available, likely in academic publication in technical journal, or equivalent. Data collected in an academic and/ or prototyping laboratory.	Not applicable
		**Feasibility**	**2–4**	**1–2**	*Can it work?*	Prototype development with all necessary system components designed and shown to meet specifications	Feasibility study data	All key risks are known and no data exist to suggest they cannot be addressed.	Feasibility study complete, and data indicate that product specifications can be met. Where relevant, manufacturing process data suggests design is manufactural reproducibly.	Development is under a quality management system; MRD and PRD ("TPP") exist in draft form.
**Late** **Development**	**Development**	**Early Development**	**5–7**	**3–4**	*Can it give* *the same* *result every* *time?*	Final system design specifications complete ("design lock") and novel manufacturing processes established.	MRD and PRD	Risk register complete under mature quality system	"Alpha" product in a mature production environment meets design specifications in laboratory testing.	MRD and PRD are final, product is under design control
		**Late Development**	**5–7**	**5–7**	*Can it be* *manufactured* *and work* *every time?*	Final commercial product produced on pilot (or final) manufacturing line	Verification data	Technical file complete other than clinical validation.	"Beta" product meets product specifications and is ready for validation. Verification plan exists and verification meets requirements.	Technical file ("dossier") begun
**Clinical Trials**	**Regulatory** **Trials**	**Validation**	**8–9**	**8–9**	*Does it work* *as intended* *in the hands* *of customers?*	Clinical testing in settings of use.	Validation study data ("regulatory trial")	Technical file for regulatory submission, including clinical data complete. Product meets specifications.	Validation study complete. Product meets specifications.	Technical file ("dossier") complete
**--**	**On Market**	**Commercialization**	**--**	**10**	*Is it a* *business?*	Customer use after regulatory clearance.	--			

Abbreviations: TRL, Technology Readiness Level; MRL, Manufacturing Readiness Level; MRD, Market Requirements Document; PRD, Product Requirements Document; TPP, Target Product Profile

### iv) Inputting the pipeline of candidates into the adapted P2I model

As described previously, we adapted P2I v.1 into P2I v.2, adding two archetypes (unprecedented vaccines, and other products) and modifying a number of the assumptions. We then used the model “prospectively”—that is, once we had determined which candidates were in the pipeline, and their target disease/condition, archetype and phase, we then inputted these candidates into P2I v.2.

For each disease and archetype, we inputted the number of candidates that were in each phase of development (for repurposed drugs, NCEs, biologics, vaccines, and other products, the phases were preclinical, phase I, phase II, or phase III; for diagnostics, the phases were concept and research, feasibility and planning, design and development, or clinical validation and launch readiness). The analysis was undertaken in 2017, and hence we chose a start date of 2017 (consistent with P2I v.1).

Based on the assumed costs, attrition rates, and time per phase for each archetype, the model estimates the costs and outcomes of moving product candidates through the pipeline *from their current phase.* When a candidate is put into in a specific phase, the model assumes that it is at the *start* of that phase and so it includes the costs of moving that candidate through to the end of its current phase.

For each archetype for each disease, the final outputs are (a) the costs of moving the archetype candidates through the pipeline from their current phase, and (b) the estimated product launches at the end of this process. For launches, we rounded only at the very end of the model. For example, for disease X, if there were 3 simple vaccine candidates at Phase II that led to 1.3 expected launches and 3 simple vaccine candidates at Phase III that led to 1.4 expected launches, we rounded the cumulative total—in this case, the total was 2.7. For this paper, we have chosen a conservative approach to presenting the launches—we have considered a launch to be a binary event, i.e., we have always rounded down (in this case, 2.7 rounds down to 2 launches). However, in Supplementary File 1, we also present the results without any rounding (e.g., in this example, 2.7 launches) and with rounding to the nearest integer (2.7 would round to 3). Both of these other approaches give less conservative estimates of the number of launches ^15^.

We did not apply a discount rate to our cost estimates. Probability of success, time of phase, cost variables, archetype and complexity classification were assumed to remain constant throughout the lifecycle of the model. We modeled only the current pipeline (i.e., we assumed that no new candidates would enter the pipeline).

### (v) Estimating the costs of priority “missing” products

As described in the Results section below, for several diseases and product types (e.g., highly efficacious vaccines for HIV, TB, and malaria), the model suggested that there would be no product launches based on the current status quo (i.e. based on the pipeline of candidates that are in the public domain). In order to estimate the costs to develop those products that are likely to be “missing” but are highly needed, we reviewed the suggested list of “important” or “game changing” diagnostics, drugs, and vaccines prioritized by the Commission on Investing in Health ( Table 5). The Commission’s list was developed through expert consensus. We examined the overlap between the Commission’s proposed products and those that our modelling suggested would still be missing (the 18 missing products are noted in Table 5). For each missing product, we used the P2I v.2 model in a retrospective manner to estimate the number of additional candidates that would be needed at preclinical phase—over and above the existing candidates—to lead to one expected launch of that product, and the associated additional cost. For example, as shown in the Results section, we found 41 HIV vaccine candidates in the pipeline and the modelling suggested that these would result in 0.49 launches. Thus, to estimate the additional costs to reach one launch, we estimated the number of additional candidates needed at preclinical phase and the associated additional costs to achieve an additional 0.51 launches (in this case, an additional 125 candidates would be needed at preclinical phase to achieve 0.51 launches, at an additional cost of $2.8 billion).

**Table 5.** Important or game changing products proposed by the Commission on Investing in Health that could help achieve a grand convergence in global health by 2035.

	Diagnostics	Drugs	Vaccines
Short term (available before 2020)			
**Important**	POC diagnostics for HIV, TB, malaria; POC viral load for HIV	New artemisinin co-formulations for malaria; new TB drug co-formulations *; curative drugs for hepatitis C; new antivirals for influenza; long-acting contraceptive implant; safe, effective, shorter duration therapy for active and latent TB; new drugs for NTDs * (with high efficacy and few side effects)	Moderately efficacious (50%) malaria vaccine; conjugated typhoid vaccine; staphylococcal vaccine; heat-stable vaccines; new adjuvants to reduce multiple dosing of vaccines; more effective influenza vaccines in elderly people
**Potentially** **game-changing**		Single encounter treatment for malaria: a one-dose cure for *vivax* and *falciparum*	
Medium term (available before 2030)			
**Important**		Antimicrobials based on a new mechanism of action	Combined diarrhea vaccine * (rotavirus, enterotoxigenic *E. coli*, typhoid, shigella); protein-based universal pneumococcal vaccine; RSV vaccine; hepatitis C vaccine *
**Potentially** **game-changing**		New classes of antiviral drugs	HIV vaccine *; TB vaccine *; highly efficacious malaria vaccine *; universal influenza vaccine

Abbreviations: POC: point-of-care; NTDs: neglected tropical diseases; RSV: respiratory syncytial virus; TB: tuberculosis *The 18 “missing” products based on modeling the current pipeline (highly efficacious vaccines against HIV, TB, malaria, and hepatitis C; combined vaccine against multiple diarrheal diseases; complex NCE for TB; NCEs for 12 NTDs (based on the WHO list of NTDs at http://www.who.int/neglected_diseases/mediacentre/factsheet/en)).

### (vi) Sensitivity analysis

As a final step, we conducted a sensitivity analysis, adopting an approach proposed by Mestre-Ferrandiz *et al.* at the United Kingdom Office of Health Economics in their study, “ The R&D Cost of a New Medicine.” We examined the impact of changing all probabilities of success per phase to 10% higher and 10% lower, and all costs per phase to 10% higher and lower. We also examined the impact of all possible combinations of these changes (e.g., 10% higher probability of success per phase *and* a 10% higher cost per phase, 10% higher probability of success per phase *and* a 10% lower cost per phase, etc.). We conducted this sensitivity analysis both for moving current candidates through the pipeline and for the costs of priority “missing” products. We did not conduct a sensitivity analysis varying the length of time per phase, because in the P2I model the length of time is independent of the cost variables (the cost parameters are per phase, not per year). We also conducted a sensitivity analysis in which we used only the assumptions from the P2I v.1 model, which helps to show the impacts of the modifications that we made to P2I v.1 when we developed P2I v.2.

While the approach of changing product development assumptions by 10% higher and lower has been used in previous studies, we recognize that achieving these changes—particularly reducing the attrition rates—may not be realistic. As Paul *et al.* note: “There is little doubt that reducing the attrition rate of drug candidates in clinical development represents the greatest challenge and opportunity for pharmaceutical R&D, and arguably for sustaining the viability of the entire industry ^16^.” Reducing costs may be more feasible, e.g. through simpler trials or improved trial management. Nevertheless, we included a sensitivity analysis to show the impact of changing the underlying assumptions on our estimates.

## Results

### The pipeline of candidates for neglected diseases

In the pipeline portfolio review, we found 685 product candidates for neglected diseases as of August 31, 2017. We excluded 147 of these from the model because (a) there was insufficient information about their development phase, (b) they were already marketed, or in a development phase that is excluded from the P2I.v2 model, or (c) there was insufficient information about the candidate to be able to classify it into an archetype. After exclusion, 538 candidates were included in the model.

Supplementary File 2 gives detailed information on these 538 candidates, showing (a) the candidate’s name, (b) the health area that it targets, (c) the development phase that it was in at the time this study was conducted, (d) the archetype to which we assigned the candidate, (e) relevant source material that guided the classification, and (f) the sponsors and collaborators conducting the study of this candidate ^17^.

**Figure 2.**
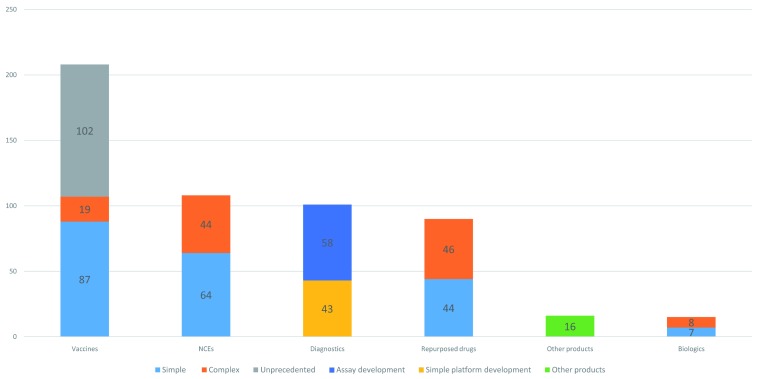
Number of candidate products for neglected diseases, by archetype.

**Figure 3.**
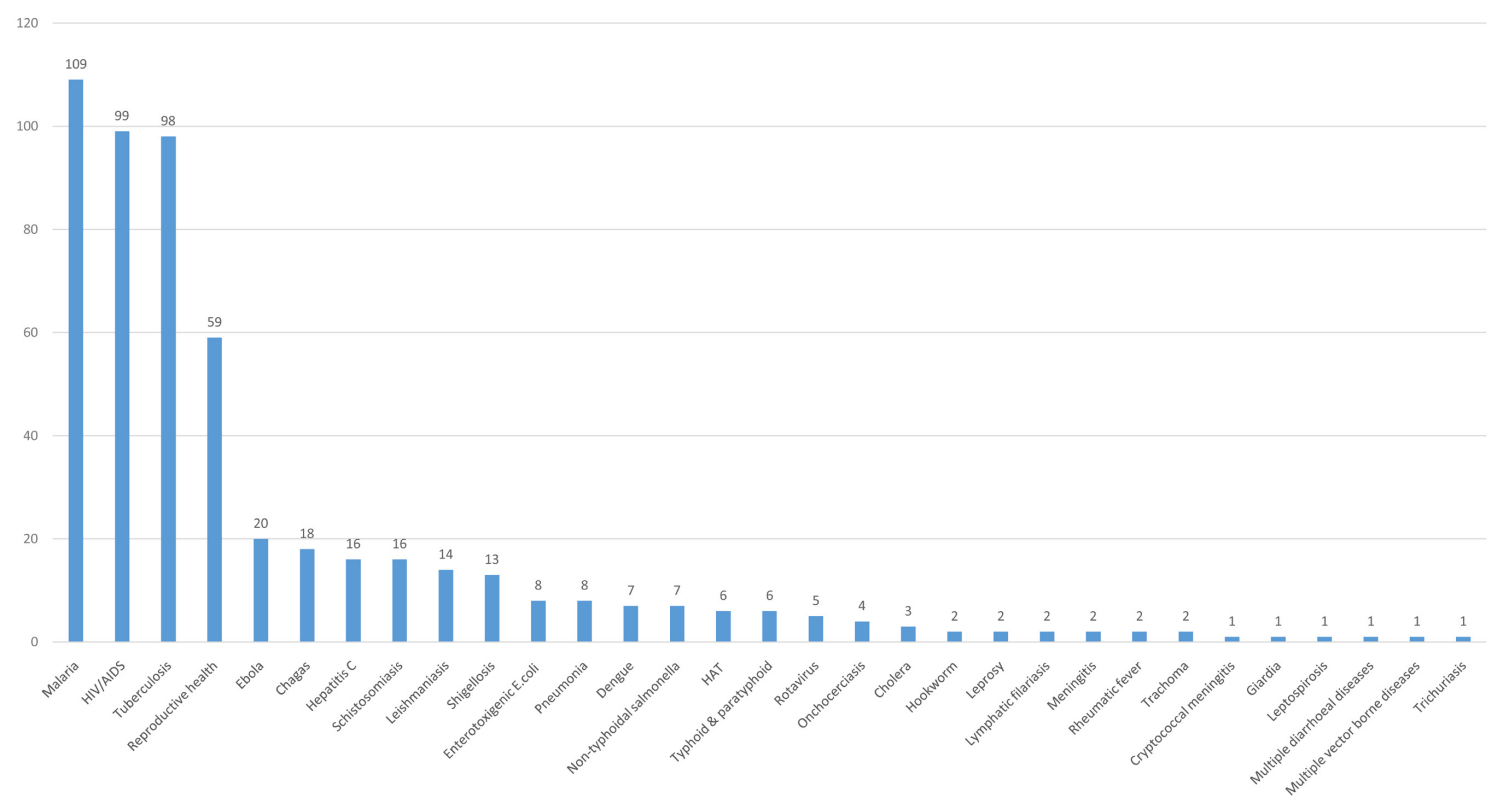
Number of candidate products for neglected diseases, by disease/condition.

Figure 2 shows the breakdown of candidates by the archetypes used in P2I v.2 (208 vaccines, 108 NCEs, 101 diagnostics, 90 repurposed drugs, 16 vector control products, and 15 biologics) and Figure 3 shows the breakdown by disease/condition. The pipeline is dominated by three diseases—malaria (109 candidates), HIV/AIDS (99 candidates), and tuberculosis (98 candidates)—which comprise nearly 6 out of 10 (57%) of all candidates. About 1 in 10 candidates (11%) are for reproductive health needs in developing countries. Table 6 shows the breakdown of candidates by disease and archetype for the 10 diseases/conditions that have the most candidates. For several health areas—particularly neglected tropical diseases—there were only one or two candidates (one candidate for cryptococcal meningitis, giardiasis, leptospirosis, multiple diarrheal diseases, multiple vector-borne diseases, trichuriasis, and two candidates for hookworm, leprosy, lymphatic filariasis, meningitis, rheumatic fever, and trachoma).

**Table 6.** Breakdown of candidates by disease and archetype for the 10 diseases/conditions that have the most candidates in the pipeline.

Disease	Biologic	Diagnostic	NCE	Vector control product	Drug Repurposing	Vaccine	Total
Malaria	0	21	30	12	8	38	109
HIV/AIDS	3	15	33	0	7	41	99
Tuberculosis	1	36	14	0	24	23	98
Reproductive Health	6	0	16	0	37	0	59
Ebola	3	0	2	0	2	13	20
Chagas	0	4	1	0	0	13	18
Hepatitis C	0	9	2	0	1	4	16
Schistosomiasis	0	2	0	0	3	11	16
Leishmaniasis	1	4	2	0	2	5	14
Shigellosis	0	0	0	0	0	13	13

### Costs to move candidates through the pipeline

Based on inputting these 538 candidates into the P2I.v2 model, the modelling suggests that it would cost about $16.3B over about 10–12 years to move all of these candidates through the pipeline ( Table 7). Supplementary File 3 gives detailed information on the breakdown of these estimated costs ^18^. As shown in Table 7, in many cases the overall costs per phase are higher for the simpler archetype (e.g., the costs in all phases are higher for simple vaccines than complex vaccines); this finding simply reflects the fact that there are more candidates of simple complexity in the pipeline (see Figure 2 and Supplementary File 2 ^17^).

**Table 7.** Costs of moving current product candidates through the pipeline from their current phase.

Archetype	Preclinical ($, millions)	Phase 1 ($, millions)	Phase 2 ($, millions)	Phase 3 ($, millions)	Total ($, millions)
Simple vaccine	301.50	88.92	561.08	4930.42	5881.92
Complex vaccine	83.00	22.62	132.35	912.93	1150.90
Unprecedented vaccine	431.60	156.62	768.77	616.45	1973.42
Simple NCE	106.60	52.33	186.92	901.48	1246.75
Complex NCE	250.00	175.60	131.37	220.39	777.37
Simple repurposed drug	-	-	185.60	469.93	655.53
Complex repurposed drug	135.00	86.35	134.22	187.24	542.81
Simple biologic	33.50	6.70	40.58	172.36	253.14
Complex biologic	66.40	6.59	46.13	217.96	337.09
	Concept & Research	Feasibility & Planning	Design & Development	Validation	
Diagnostic, assay development	6.00	26.76	91.33	180.82	304.91
Diagnostic, simple platform development	9.00	25.89	3025.46	100.31	3160.66
Other products	6.00	8.82	13.63	35.81	64.27
**Total**	1412.00	657.61	5316.86	8948.82	16348.79

**Figure 4.**
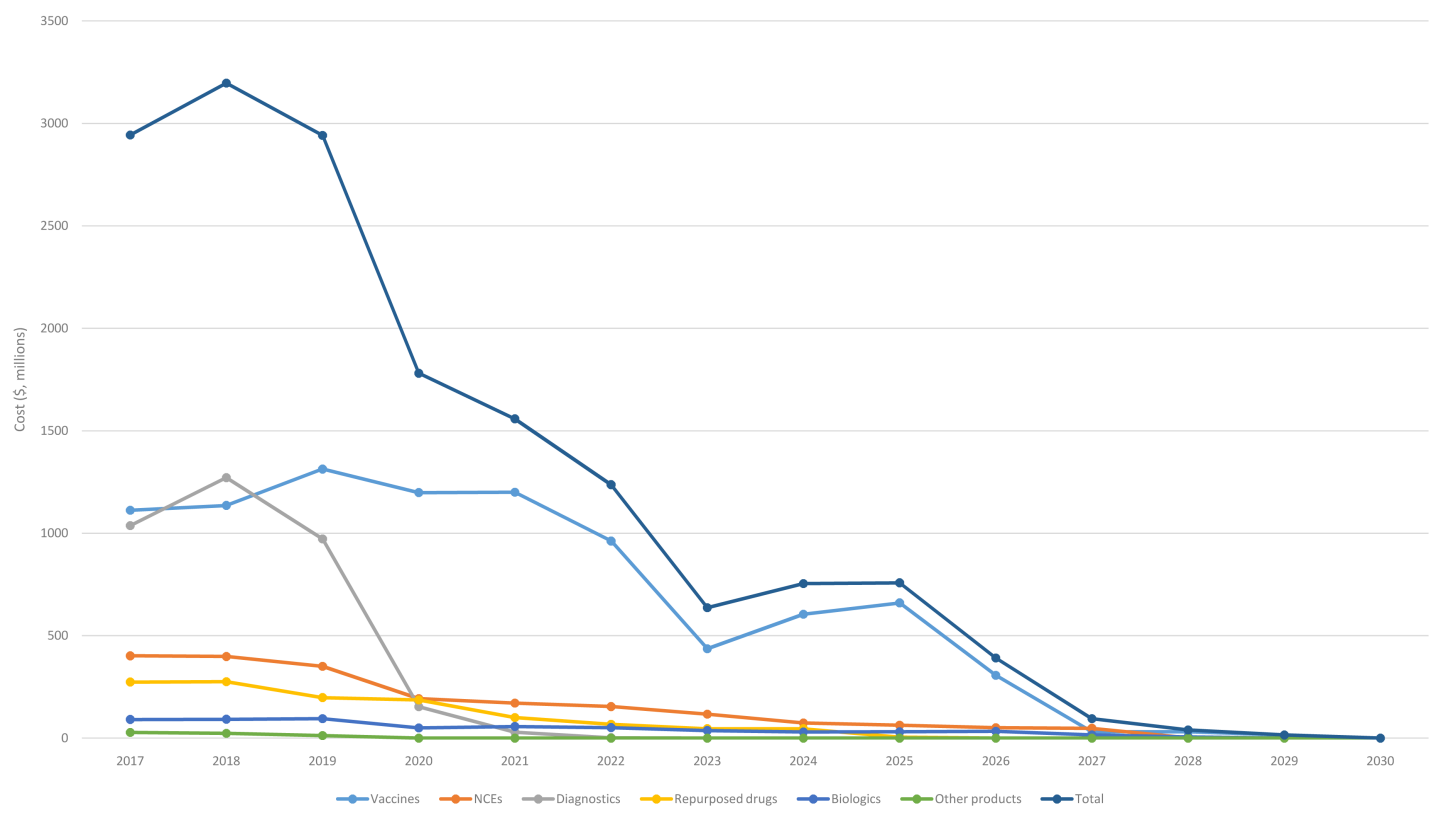
Costs over time to move candidate products through pipeline, total and by archetype.

Over half of these estimated costs (55%, $8.9B) would be for Phase III clinical trials, one third (33%, $5.3B) for Phase II trials, just under one tenth (9%, $1.4B) for preclinical development and the remainder (4%, $0.7B) for phase I trials (the percentages do not add up to 100%, since they have each been rounded). About three quarters of the costs (76%, $12.4B) would be incurred in the next 5 years assuming that all candidates are taken through all stages ( Figure 4); in reality, of course, there would be a “go/no go review” at each stage gate. The “front loading” of costs over the first five years reflects the large number of candidates at early phases and the subsequent attrition through each phase.

**Figure 5.**
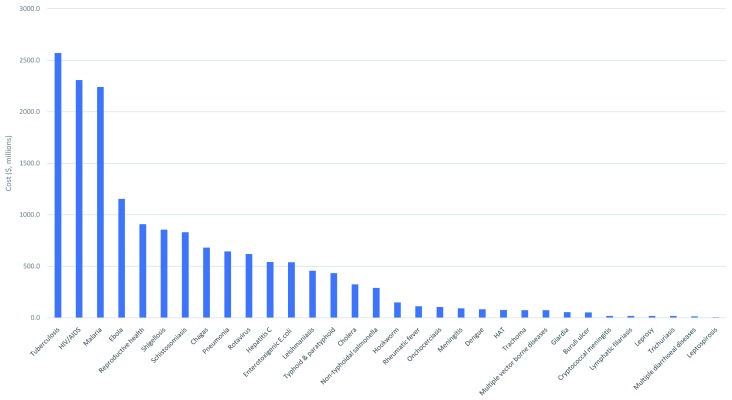
Costs to move candidate products through pipeline, by disease.

As shown in Table 7, if all the current candidates that are in the public domain were taken through all stages, over half of the development costs (55%, $9B) would be for vaccines, about one-fifth (21%, $3.5B) for diagnostics, 12% ($2B) for NCEs, about 7% ($1.2B) on drug repurposing, 4% ($0.6B) on biologics, and 0.4% (under $0.1B) on vector control products (the percentages do not add up to 100%, since they have each been rounded). The four diseases responsible for the highest costs would be TB ($2.6B), HIV ($2.3B), malaria ($2.3B), and Ebola ($1.2B) ( Figure 5).

### Expected product launches

Based on public domain information on the current portfolio of candidates for neglected diseases, and the assumptions of success at each phase for the different archetypes included in the P2I v.2 model, 128 product launches would be expected (this figure is based on rounding down the number of launches for each disease archetype; Supplementary File 1 shows the results without any rounding and also with rounding to the nearest integer) ^15^. The dominant product type for the anticipated launches would be diagnostics, which would make up almost 6 in 10 expected launches (57%, expected 73 launches), followed by repurposed drugs (13%, 16 expected launches) and simple NCEs (13%, 16 expected launches).

**Figure 6.**
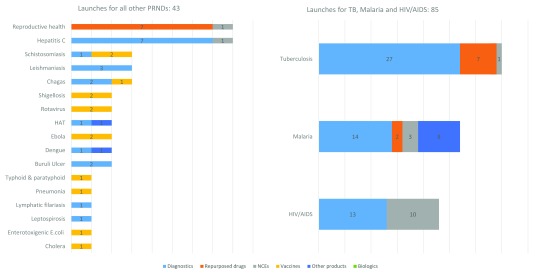
Breakdown of the portfolio of expected launches, by disease and archetype. PRNDs: poverty-related, neglected diseases

As shown in Figure 6, the model estimates that just over one quarter (27%) of all anticipated launches would be for TB: 35 launches, comprising 27 diagnostics, 7 repurposed drugs and 1 NCE. The diseases that would see the second and third highest number of expected launches, respectively, would be malaria (27 expected launches, comprising 14 diagnostics, 8 vector control products, 3 NCEs and 2 repurposed drugs) and HIV (23 expected launches, comprising 13 diagnostics and 10 NCEs).

Table 8 shows the breakdown of these anticipated 73 diagnostics launches by archetype, complexity (assay development versus simple technical platform development) and disease. For example, of the 27 anticipated TB diagnostics, 16 would be diagnostic assays and 11 would be technological platforms that enhance current technology.

**Table 8.** Breakdown of anticipated diagnostics launches by archetype (assay development versus simple technical platform development) and disease parameters, percentage change from baseline, estimated cost ($, millions), estimated number of product launches.

Disease	Diagnostic launches: assay development	Diagnostic launches: simple technical platform development	Total diagnostics
Tuberculosis	16	11	27
Malaria	7	7	14
HIV/AIDS	7	6	13
Hepatitis C	6	1	7
Leishmaniasis	3	0	3
Buruli ulcer	2	0	2
Chagas disease	1	1	2
Dengue	1	0	1
HAT (sleeping sickness)	1	0	1
Leptospirosis	1	0	1
Lymphatic filariasis	1	0	1
Schistosomiasis	1	0	1
TOTAL	47	26	73

The modelling suggests that there would be very few launches of complex vaccines (only 2). It also suggests that launches of vaccines for HIV, TB, or malaria and launches of complex NCEs would be unlikely.

Supplementary File 3 ^18^ gives detailed information on the portfolio of anticipated product launches, broken down by disease and archetype.

### Sensitivity analysis: moving current candidates through pipeline

Table 9 shows the results of the sensitivity analysis, in which we examined the impact on costs and expected product launches of changing all probabilities of success per phase to 10% higher and 10% lower, all costs per phase to 10% higher and lower, and all possible combinations of these changes. The sensitivity analysis found that the total costs to move the current pipeline through to production range from $13.4B-19.8B and the anticipated launches range from 89–160.

**Table 9.** Sensitivity analysis varying effect of changes in probability of success and cost of development per phase.

Parameters	Percentage change from baseline	Estimated cost ($, millions)	Estimated number of product launches
Baseline		16348.8	128
Probability of success	Low (-10%)	14873.3	89
	High (+10%)	17981.7	160
Average cost per phase	Low (-10%)	14713.9	-
	High (+10%)	17983.6	-
Combined	Low (-10% for both parameters)	13385.9	89
	Intermediate 1 (Cost+10%, Probability of success -10%)	16360.6	89
	Intermediate 2 (Cost-10%, Probability of success +10%)	16183.6	160
	High (+10% for both parameters)	19779.9	160

The second sensitivity analysis that we conducted in which we used only the assumptions of P2I v.1 found that the total costs to move the current pipeline through to production would be $13.4B, with 151 anticipated launches.

### Estimates of the costs of “missing” products

As shown in Supplementary File 3, based on the current known pipeline (i.e., candidates in the public domain), there are unlikely to be product launches for several diseases and product types ^18^. A comparison of these “missing” products with the list of needed priority products proposed by the Commission on Investing in Health highlights 18 missing products. As shown in Table 5, these are: highly efficacious vaccines against HIV, TB, malaria, and hepatitis C; a combined vaccine against multiple diarrheal diseases; a complex NCE for TB; and NCEs for 12 NTDs (based on the WHO list of NTDs: Buruli ulcer, Chagas disease, dengue, human African trypanosomiasis, hookworm, leishmaniasis, leprosy, lymphatic filariasis, onchocerciasis, schistosomiasis, trachoma, and trichuriasis).

Table 10 estimates the number of candidates needed at the preclinical phase, development cost, and development time to launch one product, for each different archetype, assuming an “empty” pipeline (i.e., starting product development from scratch). However, the pipeline for these 18 missing products is not currently empty; Table 11 shows the number of additional candidates that would be needed at preclinical phase—over and above the existing candidates—to lead to one expected launch of that product, and the associated additional cost. Table 11 includes the results of the sensitivity analysis (changing all probabilities of success per phase to 10% higher and 10% lower, all costs per phase to 10% higher and lower, and all possible combinations of these changes).

**Table 10.** Estimates of the number of product candidates needed at preclinical phase, development cost, and length of time until launch for to launch one product, by archetype, assuming pipeline is empty.

Archetype	Number of products needed at preclinical phase	Cost ($, millions)	Length of time until launch (yrs)
Simple vaccine	11.0	406.6	10
Complex vaccine	34.6	1057.4	13
Unprecedented vaccine	243.9	5550.0	13
Other products (vector control)	1.7	8.6	4
Simple NCE	9.5	130.3	11
Simple NCE for TB	13.1	179.8	11
Complex NCE	39.9	731.0	12
Simple repurposed drug	3.2	56.3	5
Complex repurposed drug	7.2	92.4	9
Simple biologic	11.1	299.8	11
Simple biologic for TB	14.2	381.5	11
Complex biologic	55.4	1449.8	12
Diagnostic, assay development	2.0	10.6	5
Diagnostic, simple platform development	2.6	143.6	6

**Table 11.** Estimated additional costs to launch 18 “missing” products.

Disease	Archetype	Additional number of candidates needed at preclinical phase (range *)	Additional investment needed to achieve one expected launch, $, millions (range *)
HIV/AIDS	Unprecedented vaccine	125 (85-190)	2844.4 (1740.8 – 4755.8)
TB	Unprecedented vaccine	169 (115-257)	3845.6 (2355.1 – 6432.8)
Malaria	Unprecedented vaccine	171 (117-261)	3891.1 (2396.1 – 6533)
Hepatitis C	Simple vaccine	6 (4-8)	222.2 (133.3 – 325.8)
Hepatitis C	Complex vaccine	30 (21-46)	915.6 (576.8 –1544.3)
Multiple diarrheal diseases	Simple vaccine	11 (8-17)	406.6 (266.6 – 692.4)
Multiple diarrheal diseases	Complex vaccine	35 (24-53)	1057.4 (659.2 – 1779.3)
TB	Complex NCE	27 (18-41)	495.0 (297.0 – 826.8)
12 NTDs **	Simple NCE	101 (70-155)	1851.6 (861.5 – 2331.5)
12 NTDs **	Complex NCE	478 (326-728)	8762.9 (5378.7 – 14680.6)

*The range reports the results generated by the sensitivity analysis ((changing all probabilities of success per phase to 10% higher and 10% lower, all costs per phase to 10% higher and lower, and all possible combinations of these changes). **These diseases are Buruli ulcer, Chagas disease, dengue, hookworm, human African trypanosomiasis (HAT), leishmaniasis, leprosy, lymphatic filariasis, onchocerciasis, schistosomiasis, trachoma, and trichuriasis

The total estimated additional costs (over and above the costs to move current candidates through the pipeline) to reach one estimated launch of each of these 18 missing products ranges from $13.6B to $21.8B over 10–12 years, depending on the complexity of the products ( Table 11). Around three-quarters (75%) of the costs are likely to be incurred over the first 5 years, i.e., $10.3B-16.6B. The sensitivity analysis found that the total additional costs range from $8.1B-36.6B.

Over the next 5 years, the estimated costs to move all current candidates that are in the public domain through the pipeline plus the additional costs to launch 18 missing products are about $22.7B-$29B or $4.5-5.8B per year.

## Discussion

Our study, based on data in the public domain, found 685 product candidates for neglected diseases as of August 31, 2017, of which 538 fitted archetype descriptions and could be entered into a portfolio costing model, P2I v.2. The pipeline is dominated by product candidates for HIV, TB, and malaria; of the candidates included in the model, almost 6 in 10 (57%) targeted these three diseases.

The dominance of these three diseases when it comes to product candidates is in alignment with the proportion of funding for neglected disease product development that is directed at HIV, TB, and malaria. In the 2017 G-FINDER report, which analyzes financing data for 2016, out of a total of $3.2B invested in neglected disease product development, 70% ($2.2B) was targeted at these three diseases (G-FINDER calls these “tier one diseases,” as they are in the top tier of funding) (see G-FINDER report for 2017).

In contrast, for several diseases there were just one or two candidates, reflecting much lower levels of R&D funding. For example, G-FINDER notes that a number of diseases are in the bottom funding tier (“tier three”), meaning that they each receive less than 0.5% of global funding for neglected disease product development. We found very few candidates for these diseases, e.g. just one each for cryptococcal meningitis and leptospirosis and two each for leprosy, rheumatic fever, and trachoma. The proportion of total funding directed at each disease is poorly correlated with its overall disease burden ^19^. Indeed, several high burden diseases receive very little R&D funding. For example, the Global Burden of Disease Study 2016 estimated that there were about 450 million people with hookworm in 2016 ^20^, yet it received just $3.87 million in funding for product development in that year (see G-FINDER report for 2017). In their analysis of the relationship between R&D innovation and disease burden, Barrenho *et al.* found that for neglected tropical diseases, “innovation is disproportionately concentrated in low burden diseases.”

The model suggests that moving all 538 candidates through the pipeline from late stage preclinical to launch (end of phase III clinical trials) would cost an estimated $16.3B, of which around $12.4B would be spent in the first 5 years (on average $2.5B per annum). Given this level of investment, we would expect about 128 product launches, two-thirds of which (66%) would be for HIV, TB, and malaria.

By far the largest number of launches would be for diagnostics, which are likely to make up almost 6 in 10 expected launches (57%, expected 73 launches). This high number of estimated diagnostic launches is at least partly explained by the underlying model assumptions. In particular, in the P2I v.2 model, the success rate in the design and development phase is 100% and 75%, respectively, for assay development and simple platform development, and it is 100% at the clinical validation and launch readiness phase for both assay and simple platform development. To the best of our knowledge, there have been no peer-reviewed, published studies on attrition rates through the pipeline for diagnostic development. Nevertheless, the success rates used in P2I v.2 may be overly optimistic.

Our study suggests that the current pipeline is unlikely to produce several critically needed technologies: highly efficacious vaccines against HIV, TB, malaria, and hepatitis C; a combined vaccine against multiple diarrheal diseases; a complex NCE for TB; and NCEs for 12 NTDs. This finding underscores the need for substantially scaled up resources and innovative development approaches to fill these gaps. Using the P2I v.2 model, our study estimates that the additional total cost to launch one of each of 18 prioritized “missing products” ranges from $13.6B to $21.8B, depending on the complexity of the products. Of these additional costs, about $10.3B-16.6B would be spent in the first 5 years (an annualized average of $2B-3.3B).

Thus, overall, in the first 5 years, total estimated costs to (a) move all current candidates through the pipeline and (b) develop these 18 priority missing products would be around $4.5-5.8B per year. We recognize that these two estimates were generated in somewhat different ways and so it may not be appropriate to combine them. The former was derived by “forward induction,” taking the current pipeline of candidates and projecting forwards. The latter was derived by “backwards induction,” estimating the cost of a scenario in which additional candidates are available at the pre-clinical stage—this scenario assumes additional funds will be targeted at these “missing products.” Nevertheless, we believe it is helpful to provide an aggregate estimate as it gives an idea of the overall funding gap for neglected disease product development.

How do these aggregate estimated costs compare with current spending on product development for neglected diseases? The annual G-FINDER surveys have found that the annual spending since 2008 has been around $3B, suggesting that the funding gap is at least $1.5-2.8B. There are several reasons why this is likely to under estimate the total funding need, including (i) in all likelihood, there are additional candidates in the pipeline that we did not capture in our study (because there is no information about them in the public domain), (ii) we only estimated funding needs for 18 high priority “missing products,” not *all* missing products, and (iii) as discussed below, not all costs are included.

Closing this large financing gap will require a major effort to mobilize new resources from across the public, philanthropic, and private sectors. High-income governments have been the most important source to date for financing product development for neglected diseases, but they are arguably under-investing in such research given the very large health, social, and economic returns to investment. Findings from the G-FINDER surveys suggest that middle-income countries are under-performing in terms of their overall contribution to R&D for neglected diseases, given their economic capacity and their burden of disease. The Brookings Institution’s Private Sector Global Health R&D Project has proposed ways in which private sector investments could be stimulated, such as through advanced market commitments for hookworm and schistosomiasis vaccines. Yamey and colleagues recently proposed a number of other strategies that could potentially help to close the product development funding gap ^21^. These include a health investors’ platform “to inform public, private, and philanthropic investors—and attract new investors—to fund those candidate products likely to have the largest public health benefits” and a new type of matching fund that pairs global and national resources for shared R&D priorities.

### Strengths of the study

Our study has several strengths, and we highlight two in particular. The first is its novelty. To the best of our knowledge, our study is the first to estimate the costs of global health product development from preclinical to the end of phase III based on the existing portfolio of candidates across multiple neglected diseases, and the first study to use and adapt the P2I v.1 model for this purpose. This approach complements earlier efforts to model a single therapeutic portfolio ^22^. Our approach of costing a portfolio of candidates using the current pipeline as a basis adds a different dimension to the field of global health R&D costing, and one that aligns with the way in which funders pursue a diversified portfolio of product development projects. The value of the model is in the ability to estimate costs and probable launches based on a portfolio of candidates. The model is much less reliable when it comes to predicting what will happen to *any one specific candidate*—in other words, it is prone to the ecological fallacy (making inferences about a single candidate based on data from across a portfolio of candidates).

Second, by moving beyond the costing of individual product types for specific diseases (e.g., costing only an HIV vaccine or a TB diagnostic), our study has shown more broadly—across the portfolio of neglected diseases—where the pipeline is most robust, where it is lacking, which product launches are most likely, and which products will probably still be missing based on existing candidates. For global health R&D advocates, this broad picture could potentially help to highlight critical funding and product development gaps.

### Strengths of the P2I tool

We highlight two strengths of the tool itself. First, the P2I v.1 tool that we used and adapted is available online, as are our model assumptions, model inputs and outputs, and detailed information on the portfolio review, which means that readers can replicate, improve on, and further adapt our work ^12^. The P2I v.1 tool was designed for flexibility, as shown in the way in which we adapted it, and we encourage others to refine it further. In particular, we hope that those who have access to updated, high quality data on costs, attrition rates, and cycle times will share and contribute these data to further iterations of the model. All R&D cost modelling exercises are, of course, inherently uncertain, but the steps we have taken will, we believe, allow others to “stress test” our work. In the accompanying study on the development of the P2I tool ^12^, we describe how the tool can be used to estimate health impact. We note: “The P2I model allows users to estimate the impact of a launched product on both disability, measured in disability-adjusted life years (DALYs) averted, and mortality, measured in deaths averted.” It would thus be possible to estimate the economic value of these health impacts, and therefore to estimate the rates of return on portfolio investment.

Second, most of the model assumptions were based on a large number of data points (e.g., assumptions on success rates and cycle times were based on data from of 25,000 development candidates), and were validated through examining peer-reviewed literature, industry reports/databases, and expert interviews. While no assumptions used in R&D cost models can ever be perfect, we believe that the process for developing the assumptions was “robust enough” to give realistic, real world benchmarks for costs, success rates, and cycle times per phase.

While a detailed discussion of the literature on clinical development success rates for investigational drugs is beyond the scope of this paper, our assumptions appear to be roughly in line with reported industry standards (the amount of variation differs by product types) ^23, 24^. For example, Hay *et al.* analyzed phase transitions from January 1, 2003 to December 31, 2011 in the proprietary BioMedTracker database, a database of investigational drugs, to estimate success rates per phase ^24^. For all drug indications together, they estimate that the success rate for NCEs to advance through phases 1, 2, and 3 is 64.2%, 28.6%, and 53.2%, respectively. In comparison, our assumptions on success rates were 60% (simple NCE) or 57% (complex NCE) for phase 1, 39% (simple NCE) or 20% (complex NCE) for phase 2, and 69% (simple NCE) or 40% (complex NCE) for phase 3.

### Limitations of the study

Our study has a number of limitations. First, the pipeline portfolio review only provided a snapshot at a single point in time. The pipeline is constantly changing, and between the end of our pipeline review (August 31, 2017) and today, it has already changed.

Second, our review is probably incomplete, given the lack of publicly available information on some products under development. It is particularly challenging to find information about candidates that are at the pre-clinical research phase, since studies at this phase are not included in clinical trial registries. Unfortunately, proprietary interests and non-disclosure agreements mean that we have no knowledge at all of some candidates under development. While we did our best to gain as full a picture of the pipeline as possible by using a variety of methods—such as searching databases and interviewing product developers—it is likely that we missed some candidates. Using a different search strategy or searching further databases could have identified additional candidates, such as anti-bacterial products under development (though only certain anti-bacterial candidates would qualify for the strict inclusion criteria in our study). For all these reasons, and as mentioned above, our model probably under-estimates the total costs and number of product launches. We call on the global health research community to commit to making its product development research more transparent.

A third, related limitation is that for some candidates, there is very little information on their development phase. We found it particularly challenging to obtain public data, even in trial registries, on the development phase for candidate MPTs, diagnostics, and vector control products. In some cases, we had to make a judgment call based on whatever data we could find.

A fourth limitation relates to trial capacity. We modelled the costs to launch a number of critical “missing” products—but the model does not account for whether there is the actual trial capacity to conduct the additional studies that would be needed should the additional funding be mobilized.

A fifth limitation relates to the modifications that we made to the P2I v.1 tool to create P2I v.2. As described in the Methods section, these modifications were made using a variety of data sources (see Table 2), which introduced some variation in the strength of the evidence underlying the adaptations. For example, the assumptions on probabilities of success for unprecedented vaccines in phases II and III were based on a relatively small number of data points—around 10–25 data points per estimated value. As we note in the methods section, in P2I v.2, the adjustments made to the assumptions in P2I v.1 for biologics were derived from cautious expert judgement based on early industry trends. To assess the impact of the adaptations, we conducted a sensitivity analysis in which we ran the model using only the P2I v.1 assumptions. This resulted in a lower cost estimate for moving existing candidates through the pipeline ($13.4B using P2I v.1 assumptions versus $16.3B using P2I v.2 assumptions) and a higher estimate of expected launches (151 vs. 128).

### Limitations of the P2I modelling tool

As with all modelling tools, the P2I tool that we used in this study has several limitations. First, the model is deterministic and static. It does not take into account possible improvements in product development techniques over time, such as reductions in cycle time or lowering of development costs. Historical evidence suggests that factors such as attrition rates and costs per phase do change over time, and the P2I tool does not capture such evolutions over time ^23^. We tried to address this limitation through our sensitivity analysis, but this does not fully capture the uncertainties surrounding the model assumptions. The P2I v.1 and v.2 models also do not account for down selection, in which portfolio managers decide at various stages of the development process to drop certain candidates. By examining only the current, static portfolio of candidates—and costing just these—our study does not account for future candidates that will enter the pipeline. It also does not address the minimum specifications that a product may need to meet. For instance, although the current TB diagnostic pipeline is forecast to result in several new approved diagnostics, there is no guarantee that these products would be fit-for-purpose, or offer a significant improvement over current tools.

Second, the P2I v.2 model requires users to classify every candidate into an archetype, but categorizing candidates based on the archetype definitions was challenging—especially determining a candidate’s complexity. It will be helpful for future iterations of the model to include more fine-grained, detailed descriptions.

Third, the assumptions on costs, success rates, and cycle times per phase for each archetype were based on taking averages from a large number of data points provided by industry and PDPs across a range of diseases. In the model, the averages for each archetype are the same for every disease. For example, the costs, success rates, and cycle times per phase for developing a simple vaccine for schistosomiasis are the same as those for developing a simple vaccine for hepatitis C. Thus the model does not reflect differences that there may be between diseases when it comes to product development cost structures, success rates, and cycle times. In addition, the data that we used to develop the assumptions, provided by industry and PDPs, were not just on neglected diseases—they were on development candidates for multiple infectious and non-communicable diseases, including cancer therapies. Pooling from across a huge number of data points is likely to have led to more robust assumptions, but the inclusion of data from all different types of products (e.g., cancer drugs) may have led to estimates that do not reflect neglected disease product development alone. Furthermore, for some archetypes (e.g. biologics, diagnostics, vector control products) there were few available data on development costs and success rates to inform the model assumptions; greater availability of such data would be helpful for further refining the P2I model.

Fourth, the model does not include all phases of product development R&D—it excludes the costs of early preclinical development (drug discovery, basic research) and of regulatory review and marketing authorization. Mestre-Ferrandiz *et al.*, in The R&D Cost of a New Medicine, and Di Masi *et al.* ^8^ have shown that the pre-clinical costs, including discovery, can be substantial. For example, DiMasi *et al.*’s estimate of the capitalized cost (i.e. cost including opportunity costs) of $2.6B to the point of marketing approval to develop an NCE comprises $1.1B in pre-clinical and $1.5B in clinical costs. The cost of the regulatory approval stage may represent up to 5.7% of the total R&D cost ^23^. The P2I model does not include other types of critical research that are needed to develop new products for neglected diseases, such as developing appropriate animal models, or to bring new products to poor populations, such as policy and implementation research. In addition, our estimate of costs does not include opportunity costs. There has been a contentious debate about the merits of including these costs—for example, Angell argues that cost estimates should *not* be capitalized because drug companies “are not investment houses” and they “have no choice but to spend money on R&D if they wish to be in the pharmaceutical business ^25^.” Nevertheless, the literature on R&D costs tends to include opportunity costs, and they can be large (as previously mentioned, Di Masi *et al.*’s $2.6B estimate includes $1.2B in opportunity costs).

However, we note that these are *commercial* estimates and many costs—such as the location of production—are strategic in nature and so it is much harder to derive an average cost. In the area of neglected disease R&D, the PDPs challenge these commercial costs and their own cost estimates are much lower. For example, DNDi, the Drugs for Neglected Diseases initiative, a non-profit PDP, estimates that it has spent about $39-52 million per NCE, a figure that adjusts upwards to $130-195 million when risk of failure is taken into account. Most of the supporting basic research and pre-clinical research for neglected disease R&D is publically funded as a public good and may serve many different products. Therefore, trying to develop a meaningful average cost between for-profit and not-for-profit in the later development stages was not feasible and such an average was purposefully excluded in the P2I tool. Given all of these excluded costs, the P2I model was never intended to provide a full price tag; instead, the aim was to offer the first tool to provide an evidence-based method for R&D cost comparisons where there are historical data to draw on. The intention is to inform prioritization and decision making at a global level and not to price individual product development.

It is worth repeating that the P2I v.1 and v.2 models examine the total pipeline of all candidates, with no judgements made on “go/no go” at each stage gate. In reality a portfolio would be managed and ranked with only the most promising candidates likely to be moved on to a subsequent phase. The type of decision required would be based on potential public health impact and feasibility of success. As these are products that are aimed at poor populations, where the market is largely absent, other considerations such as access and affordability will also influence the decisions on a go/no go basis ^11^.

Fifth, the model is “agnostic” when it comes to the public health value of the estimated launches—it cannot judge their clinical utility. For example, the model estimates that there could be 27 TB diagnostics (just over a fifth of all launches). This is the type of scenario that the model was intended to highlight, in order to stimulate debate about what is an appropriate approach moving forwards. Despite this high number of launches, the large unmet need for TB diagnostics may still not be addressed as almost all new developments are of an incremental rather than transformational nature. While some of the incremental developments in the pipeline might be of benefit to address different needs in different epidemiological and geographic settings, others might be redundancies. In addition, the quality and performance of the launched assays may vary widely, and therefore they may not meet public health needs. And, as mentioned, this large estimate may also reflect the underlying assumptions of high success rates in both the design and development phase and the clinical validation and launch readiness phase.

## Conclusions

This study has shown that the P2I v.1 tool is flexible enough to be adapted and used to estimate the costs and probable launches associated with moving a portfolio of current candidates for neglected diseases through the pipeline. It has pointed to gaps in the pipeline, which can be valuable in directing and prioritizing future R&D financing. It has also given an indication of the size of the financing gap, which can be helpful for future resource mobilization. Our recommendation is that other interested parties explore the use of the P2I v.1 and see how they can adapt it to create their own scenarios and share their results so that at a global level we can improve the process of supporting R&D for neglected diseases.

## Data availability

All data underlying the results are available as part of the article and no additional source data are required.

### Extended data

Supplementary file 1: Figshare: Expected launches: unrounded, rounded to nearest integer, and rounded down, https://doi.org/10.6084/m9.figshare.11836686.v1.

Supplementary file 2: Figshare: Candidates in the pipeline for neglected diseases, as of August 31 2017, https://doi.org/10.6084/m9.figshare.11835825.v1

Supplementary file 3: Figshare: Adapted P2I tool showing anticipated launches and costs by disease and archetype, https://doi.org/10.6084/m9.figshare.11836632.v1

These extended data are available under the terms of the Creative Commons BY 4.0 license ( https://creativecommons.org/licenses/by/4.0/).
