# Supplementary material for: Developing new health technologies for neglected diseases: a pipeline portfolio review and cost model
Source: Gates Open Res. 2020 Feb 19;2:23. Originally published 2018 Apr 26. [Version 3] doi: 10.12688/gatesopenres.12817.3 (PMC6139384; doi:10.12688/gatesopenres.12817.3)
Supplement: Peer review history

# Peer review: Reviewer response for version 2

**DOI:** 10.21956/gatesopenres.13939.r26621

**Published:** 2018-10-08

**Authors:** Mikel Berdud, Jorge Mestre-Ferrandiz

**Affiliations:** 1 Office of Health Economics, London, UK; 2 Independent Consultant, London, UK

After carefully reviewing authors’ responses to the comments in our referee report, we have concluded that our main comments have been addressed successfully and the requested changes have been also made. For those comments that authors were not able to make the changes, they have successfully their potential implications in the discussion section. We are keen to report that the paper is now ready for indexing.

We confirm that we have read this submission and believe that we have an appropriate level of expertise to confirm that it is of an acceptable scientific standard.

# Peer review: Reviewer response for version 2

**DOI:** 10.21956/gatesopenres.13939.r26622

**Published:** 2018-08-28

**Authors:** Lloyd Czaplewski

**Affiliations:** 1 Chemical Biology Ventures Ltd, Abingdon, UK

The authors have responded to reviewer comments.

I confirm that I have read this submission and believe that I have an appropriate level of expertise to confirm that it is of an acceptable scientific standard.

# Peer review: Reviewer response for version 1

**DOI:** 10.21956/gatesopenres.13884.r26500

**Published:** 2018-07-10

**Authors:** Mikel Berdud, Jorge Mestre-Ferrandiz

**Affiliations:** 1 Office of Health Economics, London, UK; 2 Independent Consultant, London, UK

The research article by Young and colleagues, entitled *Developing new health technologies for neglected diseases: a pipeline portfolio review and cost model*, is a research estimating; (i) the funding requirements to move products – currently at development stages – for Neglected Tropical Diseases (NTDs) through the pipeline until market launch, and (ii) the funding required to increase the R&D effort in order to ensure at least one launch for each of 18 missing priority products ranges for NTDs of greatest need. Resulting from adding up these two estimates, authors present a headline figure of $4.5B-$5.8B/year of funding requirement over the next 5 years, which is around $1.5B-$2.8B larger than the actual funding ($3B/year).

 It is an original piece of work which represents the first attempt to estimate the R&D cost required specifically for a global health strategy aimed at tackling NTDs and meet the health targets included in the UN Sustainable Development Goals (SDGs) adopted in 2015. Its originality resides in using the methodology to estimate the R&D cost of a new drug validated by the literature to estimate the cost of moving the current portfolio of candidate drugs for NTDs through the pipeline. To our knowledge, this is the first time that such empirical approach has been used to estimate the R&D investment required for global health, which typically has been estimated by comparing historical data of global R&D expenditure for NTDs with its achievements in terms of health burden and health gains.

 Research is also relevant mainly because, in a context where the R&D expenditure for NTDs has been declining steadily, it helps understand governments, policy makers, philanthropic donors and industry, not only how much additional investment effort must be done in the following years to achieve UN global health objectives, but also in which diseases (and health technologies) the effort must be specifically increased and by how much – although this latter is only partially addressed as it requires analyses on the return rate to be performed.  

 In general, we feel methods and data are appropriate. The estimate is based on a final sample of 538 product candidates covering 35 NTDs which were selected from a wider sample of 685 candidates after applying inclusion criteria. Cost estimates are calculated by using an adapted version of Portfolio to Impact financial modelling tool (P2I v.2). Adaptations included more product types and changes over assumptions to tailor it to current portfolio for NTDs. This is also an outcome of this research which opens the door to further research although adaptations should be challenged, discussed and revised. All data are publicly available, as well as the P2I model, so interested readers could, at least in theory, replicate results (we have not done it).

 We have listed up to 11 comments. Our first general comment relates to references and placing this analysis relative to previous work. Whilst literature has been quite productive estimating the R&D cost of a single new drug, authors only cite the work by DiMasi et al. (2016) ^1^. We encourage authors to read in detail the work by Schuhmacher et al. (2016) ^2^ which reviews all the relevant literature on the topic, including the literature estimating the R&D cost, development phases success rates and development times. We consider that, the reading of this work will give the authors a deeper knowledge of probabilities of success, time intervals and R&D cost of R&D phases including drug-discovery, pre-clinical and regulatory approval. The cost of the drug-discovery and the regulatory approval stages are missed in the analysis with the latter not even mentioned. This issue is addressed below. Exploring the latest estimates released in the literature, authors would be able to compare them with the ones used for the modelling in the present work for validation purposes and eventually for filling potential gaps still remaining in the analysis. Additionally, we would encourage authors too, to read Hay et al. (2014) ^3^ in detail for assumption validation. The work by Hay and colleagues presents detailed estimates of development success rates, broken down by phase, therapeutic area and archetypes. These detailed estimates should be used to validate, by comparison, authors’ assumptions for the modelling. Finally, another reference worth read carefully by authors is Mestre-Ferrandiz et al. (2012) ^4^ in which figures presented by the relevant literature until the year of its publication is listed and comparatively assessed using tables. In combination with Schuhmacher et al. (2016) this would provide a deeper knowledge of the historical evolution of what relevant literature have published on the topic of the R&D cost of a new drug including how consistent are trends showed over time by success rates, out-of-pocket costs and times of development phases.

 Our second general comment is regarding the modelling adaptations and insufficiencies. The model estimates the R&D cost in a validated way, but the analysis missed the cost of basic research, drug-discovery and, most importantly, the cost of regulatory review submission and marketing authorization that is typically included as an additional phase in the relevant literature. Authors explain why they have not been able to estimate the cost of basic research and drug-discovery stages and justify their exclusion using the argument that they are underestimating the true cost. It has been shown (Di Masi et al (2016), Mestre-Ferrandiz et al. (2012)) that pre-clinical costs can be a sizeable share of total cost, so it would be nice to have some discussion about how big, or small, these are. The underestimation argument might be also used to exclude regulatory approval cost, in case that such costs were not possible to estimate, but authors omit any explicit mention to the reason why they have not been included in the analysis. As the cost of regulatory approval stage is “standard” for all archetypes of drugs and diseases, it should not be very complex to estimate. Consequently, authors should consider its possible inclusion – or at least its omission should be explained further – as it may represent up to a 5.7% of the total R&D cost (Schumacher et al., 2016).

 Third, we feel the issue of “opportunity cost” / “cost of capital” is only discussed very briefly. This is an important issue, as raised by Mestre-Ferrandiz et al. (2012), as it can have a significant impact on the total cost of R&D. The authors, when commenting the estimate of the R&D cost of a new drug published by DiMasi et al (2016), argue that “[…] the estimate can be criticized for, among other things, including $1.2B in “time costs” (the expected returns that private investors forgo while a drug is in development).” We feel this assertion needs further discussion. We will not question whether $1.2B of opportunity cost is too much or not, but under our view, not incorporating the cost of capital into the present analysis is one of the weaknesses of this research. Mostly because the whole literature is aligned to include it to some extent. Is the measure of the cost of capital and not its inclusion which is subject to debate. 

 The fourth general point we want to make is that we are also concerned by the lack of specificity of the information used to make assumptions. Assumed probabilities of success, development times and phase specific costs for the costing modelling are not always specific for new drugs developed for NTDs. They are based on mixed sources as Table 2. shows. The majority, highlighted in orange, come from P2I v.1 modeling and are not specific for candidates for NTDs. Other, highlighted in blue and yellow, come from Risk-Adjusted Portfolio Model and B&M Gates Foundation in-house data. When comparing assumptions coming from different sources between complex and unprecedented vaccines (a new archetype), only the probability of success for phase 2 and 3 are different, being the rest of assumptions exactly equal. Authors should explain better why the reminder assumptions imply the same values for the parameters, even though they come from different sources and are based on different samples. Some assessment of why phase 2 probability of success for unprecedented vaccines is as low as 5% should be also provided. It seems to us to be very low and although the number of datapoints used for its estimation is shown in the paper, such number of datapoints result unmeaningful as reader is not aware about the number of datapoints used for the same estimate in the case of complex vaccines. The same applies – although to a lower extent – to the phase 3 probability of success. In addition, all assumptions for simple and complex repurposed drugs are equal although many of them come from different data sources too. We wonder here how sensible is to assume that for pre-clinical and phase 1 stages both have the same costs when simple repurposed drugs are assumed to have enough safety data to start development in phase 2, while complex repurposed drugs require some phase 1 clinical trials (see table 3). The reader could be confused with assumptions in table 2 and, beyond the explained differences in data sources, the article does not make clear neither the differences/equalities in assumptions coming from different sources nor their impact on the results.

 Fifth, the authors perform a sensitivity analysis to assess impact of assumptions on results. They use the same approach as Mestre-Ferrandiz et al. (2012) which consists in decrease/increase by 10% both, probabilities of success and times of development phases. Such approach allows to perform four combinations. We will not question the appropriateness of performing the same sensitivities for the present work, although it would be useful to explain how realistic it is to achieve the 10% change for each of the variables (for example, achieving a 10% reduction in time might be very complicated, but could be achieved for phase 3 attrition rate). In addition, we want to point out that sensitivities are only presented for the cost of moving product candidates through the pipeline (at least this is what the paper seems to present in page 20). Sensitivities also affect the number of additional products required per phase to ensure one launch for the 18 priority products, as well as their cost per development phase. To our understanding, such sensitivity is not presented in the paper and its impact seems to be significant on the headline figure of the paper ($4.5B-$5.8B). Why authors do not present these sensitivities should be explained or they should be included. Another sensitivity missed in the paper is one assessing impact of time of development assumptions, which is due to neglecting the cost of capital from the analysis – as mentioned above. Authors should inevitably check how much weight these assumptions have on the cost estimate, but they neglect to perform such sensitivity analysis. Finally, less important but worth to point out is that authors may consider presenting the estimate of the cost resulting by applying only the assumptions in the P2I v.1 model. The resulting figure should be compared with the one obtained by introducing the other assumptions in the modeling (i.e. RAP, BMGF).

 Sixth, the total resulting cost – the headline figure of the article – comes from adding figures from two sources: (i) the cost of moving actual current portfolio for NTDs through the pipeline and, (ii) the increased portfolio needed to ensure at least one launch for 18 priority products for NTDs. Approaches used to estimate each are opposed one to the other. While the former consists in projecting the reality (forecasting the true cost by forward induction), the latter consists in estimating the cost of a hypothetical case (forecasting by backward induction). The two estimates are presented separately. As readers we have been able to separate them clearly along the paper. However, the headline figure, the one compared to actual funding (G-FINDER) to assess the funding gap, results from adding up the two separate estimates. Question is, how sensible is to add up these to figures? For the 18 priority products, the approach assumes that increased funding will increase the number of product candidates, but additional funds may be driven to other candidates with larger expected returns. The fact that reality is showing scarcity of investment in these priority areas is showing the necessity for other incentives rather than just funding. This should be discussed more in depth in the paper, as well as authors raising this caveat about the interpretation of the aggregated figure.

 Seventh, as authors recognise, cost modelling is static. It means that assumptions over the drug development productivity (i.e. portfolio composition, costs of development phases, probabilities of success) remain unchanged along time. It is a strong assumption, as suggested by the literature (for instance, Schuhmacher et al., 2016). Attrition rates, time of development and costs per phase have been changing during the last decades due to technology changes, biomedical science advance, etc. New candidates are also expected to enter in the portfolio. Such constant evolution of the drug R&D landscape will affect the costs and funding required to tackle NTDs. One possible way to assess this impact is to analyse the historical evolution of attrition rates, time of evolution and cost of phase of development, estimate the trend and forecast to where they are going so the assumptions in the modelling could be calibrated taking into account such dynamic behavior of the pharmaceutical R&D process and technology.

 Eight, if we take a simplistic approach to estimate the cost of one product, we understand that for the $16.3B there are 128 products launched (over the next 10-12 years). Dividing one by the other, it gives $127m. Applying the same algebra for the 18 “missing” products gives a figure of circa $1bn (total cost of $13.6bn and $21.8bn). Costs differences are very significant, so would be interesting to understand in more detail the reasons for such (big) differences.

 Nine, it would be nice to see the expected product launches, year by year, to understand the evolution. Interestingly, for “missing” products, the authors say that around 75% of the costs are likely to be incurred over the first 5 years – why is that?

 Ten, there is a very nice analysis done by Barrenho and colleagues in Imperial College Business School, on “Does global drug innovation correspond to burden of disease? The neglected diseases in developed and developing countries”. The authors should see how their work differs. The paper can be found here: https://spiral.imperial.ac.uk/handle/10044/1/51581.

 Finally, and as some further research, it would be nice to understand from the authors whether some modelling could be done, based on this analysis, to estimate the rate of return of the R&D funding, and whether it differs by archetype, for instance. This information could be very useful for policy makers to help them identify how best to spend the extra dollar on new drugs for NTDs. As an illustration, some analyses can be found in:  Buxton et al., 2008 ^5^, Glover et al, 2014 ^6^).

 Other minor points worth mention are:

- It would be nice to do further analysis on the products that have been put on hold indefinitely and explore reasons for that.
- Sometimes authors justify assumptions n expert views and/or assessments, but these are not explained – page 7, bullet point 1 for instance. Our guess is that experts have a rationale to argue in favor of such assessments. This rationale is not discussed/explained in the paper. Deeper explanation will increase assumptions robustness.
- More information about excluded candidates would be welcomed by the reader. To which archetypes they belong or whether they belong to the 18 priority products or not for instance.
- One typo spotted in the heading for table 11 (page 22): a space is needed between number 18 and “.

When authors refer to costs, they are referring to “out-of-pocket” costs?

We confirm that we have read this submission and believe that we have an appropriate level of expertise to confirm that it is of an acceptable scientific standard, however we have significant reservations, as outlined above.

## References

[1] : Innovation in the pharmaceutical industry: New estimates of R&D costs. *J Health Econ*.2016;47: 10.1016/j.jhealeco.2016.01.012 20-33 10.1016/j.jhealeco.2016.01.012 26928437

[2] : A Review of the Pharmaceutical R&D Efficiency: Costs, Timelines, and Probabilities.2016; 10.1002/9783527693405.ch4 60-79 10.1002/9783527693405.ch4

[3] : Clinical development success rates for investigational drugs. *Nat Biotechnol*.2014;32(1) : 10.1038/nbt.2786 40-51 10.1038/nbt.2786 24406927

[4] : The R and D cost of a new medicine. *Monographs*.2012;

[5] : Medical research: What’s it worth? Report for the Medical Research Council, the Wellcome Trust and the Academy of Medical Sciences. *Office of Health Economics*.2008;

[6] : Estimating the returns to UK publicly funded cancer-related research in terms of the net value of improved health outcomes. *BMC Med*.2014;12: 10.1186/1741-7015-12-99 99 10.1186/1741-7015-12-99 24930803PMC4058434

# Response

**Published:** 2018-08-15

**Authors:** Gavin Yamey

Many thanks indeed to Mikel Berdud and Jorge Mestre-Ferrandiz for this very helpful review.  In the revised version of our study (version 2), we have addressed all three reviewer reports.

 Mikel Berdud and Jorge Mestre-Ferrandiz's peer review report lists 11 substantive comments, and our amendments are summarized below:

 1. **Missing literature on assumptions**: the referees kindly gave us additional examples of studies that can inform estimates of cost, attrition rate, and cycle time per phase for product development.  We now cite and briefly discuss these additional papers in the Discussion section, specifically the papers by Schuhmacher *et al*. and Hay *et al.*

 2. **The costs that are missing in our estimate**: the referees argue, and we agree, that we should be more explicit in noting which costs are missing (i.e. basic research, drug discovery, and the costs of regulatory review and marketing submission). We have now expanded our discussion of these missing costs, in the section with the sub-heading “Limitations of the P2I modelling tool.” We would also refer readers to the companion paper (reference 12 in our paper) and the original 2016 TDR report (reference 11 in our paper) for further information on the model’s development.

 3. **Opportunity costs:** as recommended by the referees, we now mention opportunity costs, again in the in the 4 ^th^ paragraph under the sub-heading “Limitations of the P2I modelling tool.”

 4. **The source of the data for the assumptions in Table 2**: The referees note that P2I.v2 uses a range of data sources as shown in Table 2 and suggest that we give more explanation of how the different sources affected the assumptions, specifically for unprecedented vaccines. We now give more explanation in the revised paper. We now also discuss—under the Limitations section of the Discussion—the limitations of our approach to modifying P2I v.1 to create P2I v.2.  

 In the revised Discussion, we state:

 “A fifth limitation relates to the modifications that we made to the P2I v.1 tool to create P2I v.2. As described in the Methods section, these modifications were made using a variety of data sources (see Table 2), which introduced some variation in the strength of the evidence underlying the adaptations.  For example, the assumptions on probabilities of success for unprecedented vaccines in phases II and III were based on a relatively small number of data points—around 10–25 data points per estimated value. As we note in the methods section, in P2I v.2, the adjustments made to the assumptions in P2I v.1 for biologics were derived from cautious expert judgement based on early industry trends. To assess the impact of the adaptations, we conducted a sensitivity analysis in which we ran the model using only the P2I v.1 assumptions. This resulted in a lower cost estimate for moving existing candidates through the pipeline ($13.4B using P2I v.1 assumptions versus $16.3B using P2I v.2 assumptions) and a higher estimate of expected launches (151 vs. 128).”

 With respect to having the same assumptions for simple and complex repurposed drugs, this reflects an attempt to be as “conservative” as possible when it comes to costs.  See also point 5 below—we have now conducted a sensitivity analysis in which we use only the assumptions from the P2I.v1 model, which helps to show the impacts of the P2I v.2 modifications based on RAP and BMGF data.

 5. **Conducting additional sensitivity analyses**: We have now conducted two new sensitivity analyses suggested by the referees, which are described in the revised Methods and presented in the revised Results:

 (a) A sensitivity analysis for the 18 “missing” products, including both the additional number of candidates needed at preclinical phase and the additional investment needed to achieve one launch.

 (b) A sensitivity analysis in which we use only the assumptions from the P2I.v1 model.

 We did not conduct a sensitivity analysis varying the length of time per phase, because in the P2I model the length of time is independent of the cost variables (the cost parameters are per phase, not per year)—we now mention this in the Methods section (under sensitivity analysis).  In response to the referees’ suggestion to reflect how realistic it would be to change our assumptions by 10% higher or lower, we now briefly mention this in the section on sensitivity analysis and cite Paul et al’s work. We would also recommend that this tool is intended to generate different scenarios by adjusting these variables.  There is no single answer but a set of outcomes within a range. This is based on historical data and will change over time as new data are added.

 6. **The appropriateness of combining the two different estimates (current pipeline versus missing products):** we now discuss the pros and cons of aggregating these two estimates in the revised Discussion section.

 7. **The static nature of the cost modeling**: we have expanded our discussion of this in the revised section on the “Limitations of the P2I tool.”

 8. **Costs per candidate**: the referees suggest taking a “simplistic approach” to estimate the cost of one product, by simply dividing the ‘portfolio cost’ by the anticipated launches (i.e., $16.3B divided by 128). As mentioned in our response to other reviewers, we do not feel this is appropriate since the P2I tool is a portfolio financial modeling tool—it is not intended to model individual product costs. 

 9. **Visualizing product launches year by year:** we are unable to produce this illustration/figure because of the nature of the rounding. As we state in the paper: “For this paper, we have chosen a conservative approach to presenting the launches—we have considered a launch to be a binary event, i.e., we have always rounded down (in this case, 2.7 rounds down to 2 launches).”  We did the rounding at the end of the modeling (rather than at each phase of the model), which precludes us from showing launches over time.  The referees ask why 75% of costs are incurred in the first 5 years—we now address this in the revision (it reflects the large number of candidates at early phases and the subsequent whittling down through each phase).  In the revised Results section, we now state: “The “front loading” of costs over the first five years reflects the large number of candidates at early phases and the subsequent attrition through each phase.”

 10. **Citing the Barrenho *et al. *study**: we now mention and cite this study in our revised Discussion.

 11. **Using the model to estimate the rate of return on R&D**: while we are unable to do this analysis for the current paper, the model could be used in this way. As mentioned in our response to Referee Report 1, we now discuss this in the revised paper.

 The referees list 4 minor points at the end of their report:

- They suggest conducting an analysis on the products that were put on hold indefinitely, exploring reasons why they were put on hold—this future analysis would be interesting, but is outside the scope of the current project.
- The role of expert views/stakeholder interviews: we have tried to address this point, as described in our response to the other reviewers.
- Information on the excluded candidates: See “Supplementary File 2. Candidates in the pipeline for neglected diseases, as of August 31, 2017.”
- Typo in the heading for Table 11: we have now fixed this.

# Peer review: Reviewer response for version 1

**DOI:** 10.21956/gatesopenres.13884.r26502

**Published:** 2018-06-22

**Authors:** Kevin Outterson

**Affiliations:** 1 Combating Antibiotic Resistant Bacteria Biopharmaceutical Accelerator CARB-X, Boston University, Boston, MA, USA; 2 Health and Disability Law, Boston University, Boston, MA, USA

This is an extension of a previously published model for R&D output for a portfolio of neglected diseases. The results give us the best available estimate to date of the costs to complete both the existing pipeline for neglected diseases and to create a pipeline designed to achieve portfolio-level output goals. These are useful tools in R&D planning for global health.

 Table 2 shows the relevant parameters for the model, such as length of phase, phase success rates, and costs. This is an important table. The process for reaching these parameters is described, but I was left with several questions: (1) How do these rows compare with the published literature? (2) What was the influence of the stakeholders in revising the parameters from the published literature reference point? (3) Did the stakeholders move the parameters in any particular direction (yielding greater or lesser expense and risk)? These parameters drive the results and how they were achieved is important. It appears that a similar model was used within the Bill & Melinda Gates Foundation to evaluate product development partnerships supported by the Foundation. If so, it would be good to know explicitly how the model was honed in response to actual experience.

 Supplemental File 1 lists the neglected diseases studied and the expected R&D outcomes based on the model. I don’t have the expertise to comment on most of these categories, but the numbers related to bacterial diseases seemed too low to me. For example, the model predicted no drugs, diagnostics or vaccines targeting multiple diarrhoeal diseases and just two vaccines (but no drugs or diagnostics) for Enterotoxigenic E.coli (ETEC). I suspected that the model input data might have missed existing clinical and preclinical projects that could conceivably target these diseases, from antibiotic R&D sources that have not traditionally been included in neglected disease databases. Examining Supplemental File 2 allowed me to spot check whether companies known to me to be pursuing such work were listed. (The CARB-X portfolio is listed at www.carb-x.org). I suspect that the authors’ data sources were heavily weighted towards the neglected diseases community and did not include more traditional antibacterial projects that are generally Type I or Type II diseases. If my hypothesis is correct, then some relatively modest changes in the results would probably occur: the researchers would find additional projects that required financial support in order to reach the market and therefore fewer entirely missing programs for the target of new classes of antibiotics and prevention against bacterial diseases.

 In future articles, I hope for updates from these authors, comparing the model predictions with actual results with regard to the parameters in Table 2, and calling out specific updates in light of actual experience. This is valuable work.

 Another portfolio-level effect might be added to a future model. A single project, progressing alone might have certain parameters on cost, time and attrition. How does that change when they are not alone, but part of a cohort of projects, often from the same group of funders, such as charitable foundations? I hypothesize that if the funders coordinate open sharing of data and information, the cohort should be more efficient, but it is also possible that groupthink could send entire research portfolios down unproductive pathways or that rapid scale up could constrain available human and technical resources, raising costs and delaying timelines.

 I also note the ongoing controversies over the cost of a new drug. Could the authors use these parameters to reverse engineer the Tufts study and arrive at a per drug cost estimate? Would be an interesting comparison to this model.

 A minor typo on Table 5: should it be “POC *viral *load for HIV?”

 Thank you for the opportunity to review this article.

I confirm that I have read this submission and believe that I have an appropriate level of expertise to confirm that it is of an acceptable scientific standard.

# Response

**Published:** 2018-08-15

**Authors:** Gavin Yamey

Many thanks indeed to Kevin Outterson for this very helpful review.  In the revised version of our study (version 2), we have addressed all three reviewer reports.

 In response specifically to Kevin Outterson's report:

 1. **The process for reaching the model parameters: **This is described in more detail in the accompanying methods paper ( https://gatesopenresearch.org/articles/2-24/v2). We now make this more explicit by stating: “A detailed description of how these assumptions were developed is given in the accompanying study on development of the P2I v.1 tool. ^12”^

 The referee asks: how do the parameters compare with the published literature?  In the revised paper, in the Discussion section, we now discuss this comparison.  We state:

 “While a detailed discussion of the literature on clinical development success rates for investigational drugs is beyond the scope of this paper, our assumptions appear to be roughly in line with reported industry standards (the amount of variation differs by product types). ^20, 21^ For example, Hay *et al.* analyzed phase transitions from January 1, 2003 to December 31, 2011 in the proprietary BioMedTracker database, a database of investigational drugs, to estimate success rates per phase. ^21^ For all drug indications together, they estimate that the success rate for NCEs to advance through phases 1, 2, and 3 is 64.2%, 28.6%, and 53.2%, respectively. In comparison, our assumptions on success rates were 60% (simple NCE) or 57% (complex NCE) for phase 1, 39% (simple NCE) or 20% (complex NCE) for phase 2, and 69% (simple NCE) or 40% (complex NCE) for phase 3.”

 The referee also asks about the influence of the stakeholders in revising the parameters (asking: did they move the parameters in a particular direction?). A similar question was posed in Referee Report 3 (see our response to Report 3, item 4). This is a very good question, and we now address it in the revised paper.  As described in the paper, the assumptions were based primarily from an industry R&D cost sourcebook (e.g., assumptions on attrition rates and cycle times at each phase were initially based on a review of the attrition rates and cycle times of more than 25,000 development candidates in this source book).  The stakeholder interviews were used for validation. The interviews confirmed the assumptions and did not have a significant impact in terms of changing them. As discussed above, the interviews did highlight many of the missing costs in the model. For example, stakeholders noted that the P2I model misses many industry costs, e.g. the costs of the regulatory expenses, depreciation of infrastructure, marketing costs, etc.

 3. **Missing candidates: **we acknowledge that our pipeline portfolio review is likely to have missed candidates under development for the 35 neglected diseases/conditions, and we discuss this in our paper. The referee notes in particular that we may have missed anti-bacterial projects; in the revision, in the Discussion section, we now make this point.  Under Limitations, we now state: “Using a different search strategy or searching further databases could have identified additional candidates, such as anti-bacterial products under development (though only certain anti-bacterial candidates would qualify for the strict inclusion criteria in our study).”

 It is important to emphasize that the reason that the number of pipeline candidates for bacterial diseases is lower than the referee expected is primarily due to the strict inclusion and exclusion criteria used, rather than being an oversight. For example, the reason that no drug candidates targeting Enterotoxigenic E.coli (ETEC) ETEC were included in the portfolio is that this category is explicitly excluded from the scope of G-FINDER, and therefore of the portfolio inputs.

 4. **Future work: **The referee has a number of excellent suggestions for future studies and updates that we could provide (e.g. comparing model predictions with actual results, and examining “cohort effects” in which funders share a cohort of projects). We hope to be able to conduct such studies in the future.

 5. **Reverse engineering the cost of a new drug using our portfolio costs**. Two referee reports (Reports 2 and 3) suggested that we do this, i.e. they suggest we divide the ‘portfolio cost’ by the anticipated launches (i.e., $16.3B divided by 128). However, we do not feel this is appropriate since the P2I tool is a portfolio financial modeling tool—it is not intended to model individual product costs.  Also, the estimate of $16.3B relates to moving the current portfolio of candidates through the pipeline, some of which are currently at Phase 3. 

 6. **Typo in Table 5. **We have fixed the typo in the revised version.

# Peer review: Reviewer response for version 1

**DOI:** 10.21956/gatesopenres.13884.r26444

**Published:** 2018-05-15

**Authors:** Lloyd Czaplewski

**Affiliations:** 1 Chemical Biology Ventures Ltd, Abingdon, UK

Developing new health technologies for neglected diseases: a pipeline portfolio review and cost model by Young et al., is a well written and comprehensive review of the pipeline and an attempt to estimate the cost of delivering Sustainable Development objectives set by the UN. So this is an important piece of work and the headlines from the Abstract will be widely used and reported.

 Considerable skill and effort led to the identification of 538 products with sufficient available information to include in their cost and launch analysis. The process for project identification was clearly described although it would have been helpful to have provided the search terms used so that others could reproduce the search or update it in the future.Value is added by identifying gaps in the pipeline and estimating the costs required to support the preclinical and clinical development to launch of 18 high value products. No estimate of the cost of the fundamental research and drug discovery activity required to generate projects to feed this pipeline was provided. 

 All 538 products went into the analysis but portfolio management involves decisions about which projects to fund and a sense of prioritisation. It is not clear that the analysis provided is useful to inform policy makers about what level of funding is realistically needed to attempt to meet the UN objectives to reduce the impact of neglected diseases. 

 It is interesting that WHO's CEWG estimate of a $6 bn annual budget based on a doubling of current funding is fairly close the papers conclusion that $5 bn is required for the next 5 years to gain portfolio momentum.

 The Portfolio to Impact (P2I) spreadsheet was updated to reflect neglected diseases and more product types were added to include vector control products. The P2I model does not use costs related to basic research through lead optimization; chemistry, manufacturing, and controls (CMC); good manufacturing practice (GMP); manufacturing build up and scale-up costs; regulatory or registration fees (post-phase III); and all post-market commitments (e.g., phase IV pharmacovigilance studies). Most of these costs are project specific and therefore difficult to model but they could represent significant costs leading to an under-estimate of costs particularly preclinical costs in the first 5 years. 

 Many of the references are papers in academic literature and it is not clear what level of diligence has gone into assessing the feasibility and potential of each project.

 The diseases with most projects are HIV/AIDS 84, Malaria 88, Reproductive Health 59 and TB 62.

 Instead of funding all 293 projects in this group, if the top 20% of projects in each disease were selected by an expert funding panel, they could provide focus and reduce the number of therapeutic and vaccine projects from 421 to 186 which is a 56% reduction. This may be a more realistic start point for a portfolio assessment. 

 The world may not need 13 new HIV/AIDS or 14 new Malaria therapeutics or vaccines asap. It needs some now and some later.

 So the analysis is informative but does not use likely portfolio management to build a case for what funding would actually be needed to deliver.

 Does it make sense to proposed funding to bring 27 TB diagnostics to market? How many are needed? This is commented on in the limitations – some common sense around how many diagnostics are need would be helpful.

 This reviewer is slightly confused by Table 7 which seems to be at odds with Ref 12. The costs of a complex project at each stage of development are most likely more than the costs of a simple project yet the costs of a complex vaccine or NCE seem to be less than the costs of a simple version. The authors may wish to check this or at least help the reader understand why.

 In several places the authors describe the costs associated with moving all 538 candidates through the pipeline. It would be helpful to make project attrition clear as not all 538 projects will progress through each stage using their estimated success rates. It is not obvious that the stated project success rates have been applied to projects.

 Would it be possible to estimate what the return on investment is with respect to vaccines, therapeutics, diagnostic and others? Where is the best value spend? Preventative measures and fund those first?

 The take home numbers in the abstract may not be helpful as the lack of portfolio management, recognition of attrition, everything started in year one rather than some sense of planning and phasing means its not overly useful.

 If the suggested level of funding was available, is there adequate capacity and infrastructure in R&D and in clinical trial sites to be able to deliver? Where is the pinch point? What would the consequence of staggering the start of projects?

 The authors have provided an excellent database of neglected disease projects but may find that additional attention to portfolio project management may yield a model for future funding that reflects a deliverable reality.

I confirm that I have read this submission and believe that I have an appropriate level of expertise to confirm that it is of an acceptable scientific standard, however I have significant reservations, as outlined above.

# Response

**Published:** 2018-08-15

**Authors:** Gavin Yamey

Many thanks indeed to Lloyd Czaplewski for this very helpful review.  In the revised version of our study (version 2), we have addressed all three reviewer reports.

 In response specifically to Lloyd Czaplewski's report:

 1. **Additional details on candidate search:** The referee asked for additional details of the candidate search—we have now added a sentence to direct readers to more information.

 2. **Portfolio management**: The referee notes that “portfolio management involves decisions about which projects to fund” and later in report 1, the referee again refers to the importance of portfolio assessment. We agree, and we already note this in multiple places in the paper. In the Discussion, we state:

 “It is worth noting that the P2I v.1 and v.2 models examine the total pipeline of all candidates, with no judgements made on “go/no go” at each stage gate. In reality a portfolio would be managed and ranked with only the most promising candidates likely to be moved on to a subsequent phase. The type of decision required would be based on potential public health impact and feasibility of success. As these are products that are aimed at poor populations, where the market is largely absent, other considerations such as access and affordability will also influence the decisions on a go/no go basis.”

 We also state: “The P2I v.1 and v.2 models also do not account for down selection, in which portfolio managers decide at various stages of the development process to drop certain candidates.”

 3. **Missing costs**: The referee suggests that we should further discuss the missing costs.  Referee report 3 made the same point. We have now made a substantial addition to the Discussion in which we go into much more detail about the missing costs and we give an indication of the likely size of these missing costs.

 The substantial revision now states:

 “Fourth, the model does not include all phases of product development R&D—it excludes the costs of early preclinical development (drug discovery, basic research) and of regulatory review and marketing authorization. Mestre-Ferrandiz *et al.*, in The R&D Cost of a New Medicine, and Di Masi *et al.* ^8^ have shown that the pre-clinical costs, including discovery, can be substantial. For example, DiMasi *et al.*’s estimate of the capitalized cost (i.e. cost including opportunity costs) of $2.6B to the point of marketing approval to develop an NCE comprises $1.1B in pre-clinical and $1.5B in clinical costs. The cost of the regulatory approval stage may represent up to 5.7% of the total R&D cost. ^20^  The P2I model does not include other types of critical research that are needed to develop new products for neglected diseases, such as developing appropriate animal models, or to bring new products to poor populations, such as policy and implementation research. In addition, our estimate of costs does not include opportunity costs. There has been a contentious debate about the merits of including these costs—for example, Angell argues that cost estimates should *not* be capitalized because drug companies “are not investment houses” and they “have no choice but to spend money on R&D if they wish to be in the pharmaceutical business.” ^22^ Nevertheless, the literature on R&D costs does tend to include opportunity costs, and they can be large (as previously mentioned, Di Masi *et al.*’s $2.6B estimate includes $1.2B in opportunity costs).

 However, we note that these are *commercial *estimates and many costs—such as the location of production—are strategic in nature and so it is much harder to derive an average cost.  In the area of neglected disease R&D, the PDPs challenge these commercial costs—their own cost estimates are much lower. For example, DNDi, the Drugs for Neglected Diseases initiative, a non-profit PDP, estimates that it has spent about $39-52 million per NCE, a figure that adjusts upwards to $130-195 million when risk of failure is taken into account.   Most of the supporting basic research and pre-clinical research for neglected disease R&D is publically funded as a public good and may serve many different products. Therefore, trying to develop a meaningful average cost between for-profit and not-for-profit in the later development stages was not feasible and such an average was purposefully excluded in the P2I tool. Given all of these excluded estimated costs, the P2I  model was never intended to provide a full price tag; instead, the aim was to offer the first tool to provide an evidence-based method for R&D cost comparisons where there are historical data to draw on. The intention is to inform prioritization and decision making at a global level and not to price individual product development.

 It is worth repeating that the P2I v.1 and v.2 models examine the total pipeline of all candidates, with no judgements made on “go/no go” at each stage gate. In reality a portfolio would be managed and ranked with only the most promising candidates likely to be moved on to a subsequent phase. The type of decision required would be based on potential public health impact and feasibility of success. As these are products that are aimed at poor populations, where the market is largely absent, other considerations such as access and affordability will also influence the decisions on a go/no go basis ^11^ .”

 4. **References**: the referee notes that “many of the references are in academic literature.”  We have now added extra references that come from credible published industry experiences, where available. For example, see the new reference 20:

 Schuhmacher A, Gassmann O, Hinder M. A Review of the Pharmaceutical R&D Efficiency: Costs, Timelines, and Probabilities. In: Value Creation in the Pharmaceutical Industry: The Critical Path to Innovation. Edited by Schuhmacher A, Hinder M, Gassmann O. 2016 Wiley‐VCH Verlag GmbH & Co. KGaA.

 We would also reiterate that the basis for the model is those data reported in the PAREXEL Biopharmaceutical R&D Statistical Sourcebook – which is the industry standard for recording costs in the stages of R&D we focus on.

 5. **TB diagnostics: **The referee asks: “Does it make sense to proposed funding to bring 27 TB diagnostics to market? How many are needed?”  We now address this question in more detail in the revised Discussion. We now state:

 “Fifth, the model is “agnostic” when it comes to the public health value of the estimated launches—it cannot judge their clinical utility. For example, the model estimates that there could be 27 TB diagnostics (just over a fifth of all launches). This is the type of scenario that the model was intended to highlight, in order to stimulate debate about what is an appropriate approach moving forwards. Despite this high number of launches, the large unmet need for TB diagnostics may still not be addressed as almost all new developments are of an incremental rather than transformational nature. While some of the incremental developments in the pipeline might be of benefit to address different needs in different epidemiological and geographic settings, others might be redundancies. In addition, the quality and performance of the launched assays may vary widely, and therefore they may not meet public health needs. And, as mentioned, this large estimate may also reflect the underlying assumptions of high success rates in both the design and development phase and the clinical validation and launch readiness phase.”

 6. **Confusion about Table 7**: These costs reflect the costs of moving existing candidates through the pipeline. Therefore, the reason why the costs in all phases are higher for simple vaccines than complex vaccines, and why the costs in phases 2 and 3 are higher for simple NCEs than complex NCEs, is that there are more simple vaccine candidates and simple NCEs in these phases in the pipeline (see Figure 2 and Supplementary File 2).  We have now added a sentence in the results to clarify this point:

 “As shown in Table 7, in many cases the overall costs per phase are higher for the simpler archetype (e.g., the costs in all phases are higher for simple vaccines than complex vaccines); this finding simply reflects the fact that there are more candidates of simple complexity in the pipeline (see Figure 2 and Supplementary File 2).”

 7. **Project attrition**: the referee requests information on project attrition—Supplementary File 3 shows project attrition across the portfolio.

 8. **Estimating the returns on investment (ROI)**: the referee asks if we can generate these estimates (referee report 3 also asks about the possibility of generating an ROI). While doing so is outside the scope of this study, in the revised paper we now note that the P2I tool would potentially allow readers to do this.  We now state:

 “In the accompanying study on the development of the P2I tool, ^12^ we describe how the tool can be used to estimate health impact. We note: “The P2I model allows users to estimate the impact of a launched product on both disability, measured in disability-adjusted life years (DALYs) averted, and mortality, measured in deaths averted.” It would thus be possible to estimate the economic value of these health impacts, and therefore to estimate the rates of return on portfolio investment.”

 9. **Trial capacity**: the referee asks whether there would be adequate capacity to actually deliver on increased funding, should such funding become available.  The P2I model adjusts for this by estimating the percentage uptake and capacity for development.  This was set at 100% in this study i.e. an assumption that all products would be treated equally and moved through all stages of development. A setting of 50% would model for only half of available products being developed.   However, the reality is that this model could only inform what shape a portfolio would take but would not replace the qualitative and strategic decisions needed about go/no-go decisions. 

 In the revised paper, to address the referee’s point, we state:

 “A fourth limitation relates to trial capacity. We modelled the costs to launch a number of critical “missing” products—but the model does not account for whether there is the actual trial capacity to conduct the additional studies that would be needed should the additional funding be mobilized.”
